# Development and feasibility of an mHealth intervention for psychoeducational support of Nigerian women diagnosed with breast cancer undergoing chemotherapy: A pilot randomized controlled trial

**DOI:** 10.1371/journal.pone.0314365

**Published:** 2024-11-25

**Authors:** Oluwadamilare Akingbade, Ka Yi Hong, Oluwabukola Sharon Ayo, Adetutu Sefinat Alade, Moshood Akinwumi Lawal, Ibironke Elizabeth Somoye, Victoria Adediran, Olamide Sado, Ka Ming Chow

**Affiliations:** 1 The Nethersole School of Nursing, Faculty of Medicine, Chinese University of Hong Kong, Hong Kong (SAR), China; 2 Faculty of Nursing, University of Alberta, Edmonton, Canada; 3 Institute of Nursing Research Osogbo, Osogbo, Osun State, Nigeria; 4 Department of Nursing, Lagos State University Teaching Hospital, Ikeja, Lagos State, Nigeria; 5 Department of Nursing, Lagos University Teaching Hospital, Idi-Araba, Lagos, Nigeria; Universiti Kebangsaan Malaysia Faculty of Medicine: Hospital Canselor Tuanku Muhriz UKM, MALAYSIA

## Abstract

**Background:**

Breast cancer (BC) remains a significant health burden globally, with high incidence and mortality rates, particularly in Nigeria. Chemotherapy, a common treatment modality for BC, often leads to various physical and psychological side effects, impacting patients’ quality of life. Despite the growing use of mobile health (mHealth) interventions to provide psychoeducational support, there is a paucity of evidence regarding their feasibility and acceptability among Nigerian women with BC.

**Objective:**

To develop and investigate the feasibility and acceptability of a mHealth psychoeducational intervention (mPEI) named the ChEmo Nurse Breast cancer Application (CENBA) programme.

**Methods:**

A multi-centre, assessor-blinded, parallel-group pilot randomised controlled trial (RCT) was conducted at Lagos State University Teaching Hospital (LASUTH) and Lagos University Teaching Hospital (LUTH). Thirty women newly diagnosed with BC and undergoing chemotherapy were randomly assigned to an intervention or a control group. The intervention group received the CENBA programme, which included BC education, coping skills training, a discussion forum, and nurse-led consultations, delivered via a mobile application and phone calls over six weeks. The control group received standard care. Feasibility was assessed through consent, attrition, and completion rates, while acceptability was explored via qualitative interviews.

**Results:**

The completion rate was 93.3%. Qualitative data indicated that participants found the intervention beneficial, particularly appreciating the educational content and the emotional support provided through the discussion forum and nurse consultations.

**Conclusion:**

The CENBA programme was perceived as a feasible and acceptable mHealth intervention for providing psychoeducational support to Nigerian women with BC undergoing chemotherapy. These findings suggest that the CENBA programme could be a valuable tool in addressing the psychoeducational needs of this population, warranting further investigation in a full-scale RCT.

**Trial registration:**

This manuscript reports a feasibility study preceding the full trial, which was registered with the United States Clinical Trials registry (number NCT05489354).

## Introduction

Breast cancer (BC) accounted for one in eight cancer cases worldwide in 2020 [1]. BC is the leading female malignancy in Africa, with 168,690 cases and 74,072 deaths in 2018. Nigeria had 124,815 BC cases and 78,899 deaths in 2020 [2, 3]. Various treatment modalities exist in the management of BC. They include surgery, chemotherapy, radiation, and hormone therapy [4, 5].

Chemotherapy is used worldwide, before and after surgery, and its usage cuts across all cancer stages [6–9]. Usually, various physical and psychological side effects accompany chemotherapy usage. The physical side effects include nausea, vomiting, constipation, diarrhoea, poor appetite, and sore throat, while psychological concerns include symptom distress, anxiety, fear, deteriorated quality of life, inadequate social support, and unmet supportive care needs [10–12]. It has been estimated that 30–50% of women diagnosed with BC have psychological concerns somewhere along their illness trajectory [12, 13]. Furthermore, self-efficacy in coping, which refers to an individual’s belief in her capacity to cope effectively with or adjust to a particular situation, has been reported to reduce significantly in this population [14–16].

In addition to psychological concerns experienced by this population, educational needs have also been reported. Psychoeducational interventions (PEIs), defined as activities that combine the transfer of an organised body of information through education with supportive psychological activities, have been suggested as a means to address the psychoeducational needs of women with BC receiving chemotherapy [17, 18]. PEIs are beneficial in addressing various psychological issues among women [19, 20].

The usage of mobile phones is universal. Due to the ubiquity of mobile phones, mHealth has been recommended as an effective way to deliver PEIs [21]. Although the COVID-19 pandemic adversely impacted the diagnosis and treatment of BCs, mHealth has been adopted to support oncology patients in Nigeria during the pandemic, but evidence to support this is lacking [22, 23]. Evidence suggests that BC is among the cancers for which mHealth interventions are mostly used [21]. Mobasheri et al. [21] reviewed 185 mobile apps used along the BC illness trajectory and reported that most of the apps were not based on evidence, lacked the involvement of healthcare professionals and had safety concerns.

Similarly, Jongerius et al. [24] conducted a systematic review of 29 research-tested apps used in the care management of women with BC and reported various benefits in the management of women with BC receiving treatment. However, the review found conflicting results in the psychological dimensions, such as social support, anxiety, and depression. It recommended that more studies be conducted to test the effects of mHealth interventions on the psychological issues of women with BC.

Research-tested mHealth interventions for addressing psychological concerns of women diagnosed with BC receiving chemotherapy are relatively new, limited, and not yet popular. A systematic review of mHealth interventions used among women with BC receiving chemotherapy revealed that mHealth interventions could significantly reduce symptom prevalence, symptom severity, and supportive care needs, and improve self-esteem, emotional functioning, and quality of life [25]. Similarly, the systematic review reported that most previous mHealth interventions used for this population were psychoeducational in focus. Furthermore, four key components–BC education, group interaction, self-reporting of symptoms, and expert consultation–were found in this study. Additionally, nurses–who made up the majority of the intervention providers–are essential for providing mHealth interventions, as their involvement in the interventions significantly improved some of the psychological outcomes in this population [25]. However, none of these studies was conducted in Africa.

Furthermore, a qualitative study among Nigerian women with BC undergoing chemotherapy examined the feasibility of mHealth interventions for psychoeducational support [26]. The study identified a significant gap: the lack of a mobile app that provides information on locally available nutritious diets and other resources available in Nigeria for supporting women with BC while receiving chemotherapy [26]. This finding underscores the need to develop a new mobile app tailored to support this specific patient population. This study is aimed at investigating the feasibility and acceptability of the mHealth psychoeducational intervention (mPEI) among women diagnosed with BC receiving chemotherapy in Nigeria.

## Methods

### Study design

The study is a multi-centre, assessor-blinded parallel-group pilot RCT. This parallel-group design involves randomly allocating participants to either an intervention or a control group in a 1:1 ratio. The study was undertaken at the oncology centres of Lagos State University Teaching Hospital (LASUTH), Ikeja and Lagos University Teaching Hospital (LUTH), Idi-Araba, Nigeria. These are tertiary hospitals in Lagos, Nigeria, where advanced oncology services are rendered.

### Participants

According to Julious [27], a sample size of 12 in a group is sufficient for conducting a pilot study to test the feasibility of an intervention. Thirty-four women diagnosed with BC receiving chemotherapy from two tertiary hospitals in Lagos State, Nigeria, were assessed for eligibility. Thirty women agreed to join the study and were evenly randomised. The inclusion criteria were females (1) aged 18–70; (2) newly diagnosed with BC within the recent three months; (3) currently receiving chemotherapy; (4) who have access to a smartphone and internet; (5) able to read and write in English; (6) cognitively capable of completing the questionnaires; (7) who consent to join the study. Those women with concurrent physical or mental illness and cognitively impaired were excluded from the study. Participants for this study were recruited in June 2022 and received the intervention between July 1, 2022, and August 12, 2022.

### Intervention

**Fig 1:**
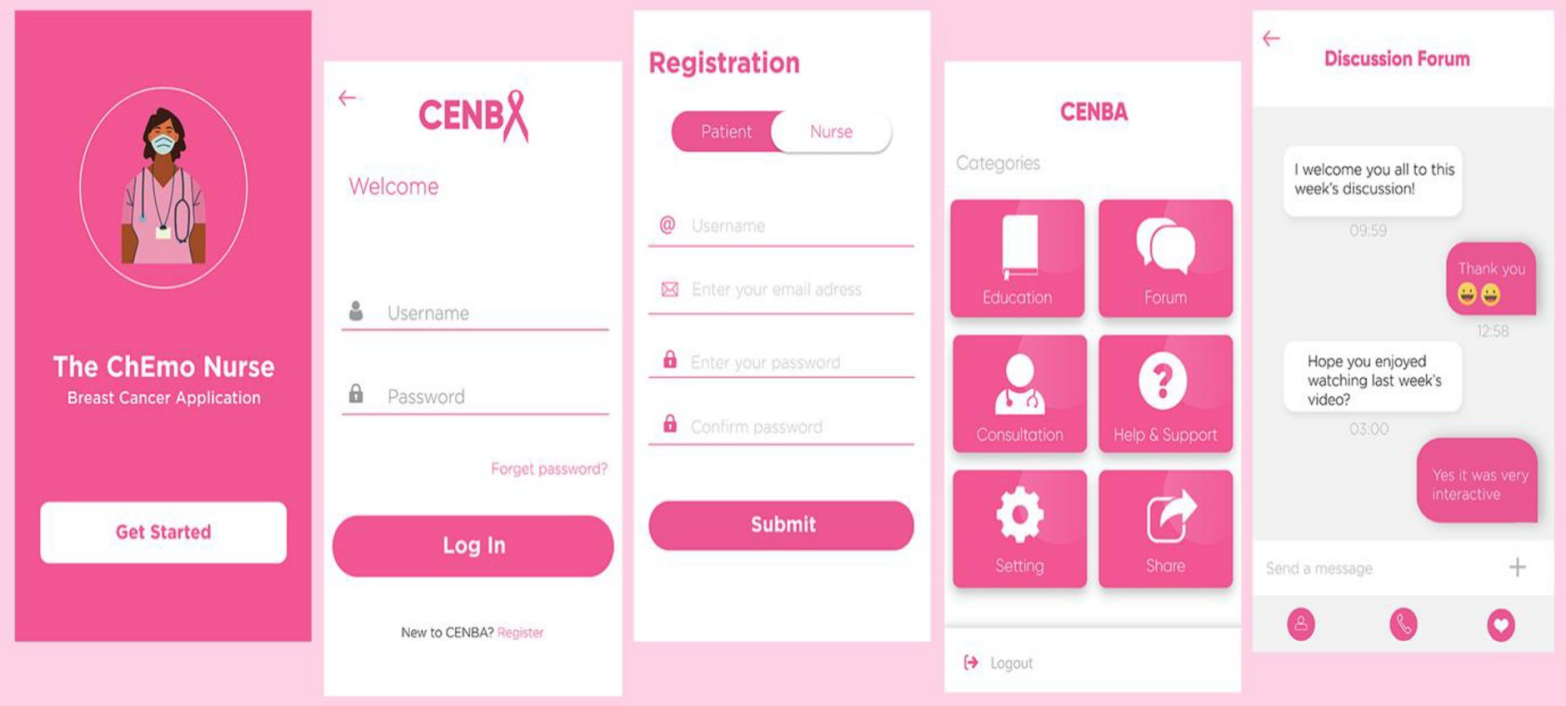
The CENBA design.

The mPEI in the study was named the ChEmo Nurse Breast cancer Application (CENBA) programme. The CENBA programme was delivered via a mobile application (app) and phone calls. The mobile app was developed in Nigeria by a research team that comprises a PhD Nursing Candidate, a nursing professor who specialised in oncology, a community health nurse, a clinical psychologist, and four oncology nurses from two tertiary hospitals in Nigeria that offer advanced oncology services–Lagos State University Teaching Hospital and Lagos University Teaching Hospital. The mobile app was developed by *Deltree Technologies*, an Information and Communication Technology (ICT) company based in Nigeria (Fig 1).

As the CENBA programme is a complex intervention with various interacting components, the development of the intervention was guided by the Medical Research Council (MRC) framework. This framework, first published in 2000, guides the development and evaluation of complex interventions [28]. A ‘complex intervention’ is an intervention with various components that interact to produce the desired outcome. According to the MRC framework, the development and evaluation of a complex intervention require four phases, which include 1) development, 2) feasibility and piloting, 3) evaluation, and 4) implementation [29]. This pilot study targets the first two phases of the MRC framework: development, feasibility and piloting. In the first phase of the MRC framework, we identified the evidence base and theory through a systematic review, while the modelling process and outcomes were achieved through the qualitative study. The second phase of the framework was achieved through the pilot study conducted to test the feasibility of the intervention.

The mobile app in the CENBA programme is supported on Android phones and was programmed with Android Studio, an integrated development environment. The programming language used is Java. The background management system was written using PHP (Hypertext Preprocessor) and Java script language with Visual Studio code.

#### Identification of the evidence base for the CENBA programme development

Evidence for the design of the CENBA programme was obtained from four sources. First, we conducted a systematic review to ascertain various mHealth interventions used among this population, the components to be included in such interventions, and the effects of mHealth interventions in addressing psychoeducational concerns in this population. Nurses were the majority of the intervention providers in the review, and they delivered the intervention using mobile apps. We also found that components of the interventions should include BC education, discussion forum, nurse consultation and psychological support. The interventions effectively reduced symptom distress, supportive care needs, and symptom prevalence and improved quality of life, self-esteem and emotional functioning among women with BC. Similarly, the intervention provided educational support for them [25].

Another piece of evidence to guide the design of the CENBA programme was obtained through a qualitative study among Nigerian women diagnosed with BC undergoing chemotherapy. The study aimed to identify their needs and understand their perception of the usefulness of the mHealth intervention. Participants interviewed in the study suggested that mHealth PEI should include information on chemotherapy, its side effects, locally available food, and exercise [26].

Additionally, we consulted oncology experts in BC management and psychoeducation for guidance on the programme’s contents. Similarly, the oncology nurses also attended training by a clinical psychologist on delivering an online psychoeducational intervention.

#### Theoretical framework underpinning the CENBA programme

The importance of theory-guided interventions has been identified in the literature. Theory-guided interventions help to understand complex phenomena by providing tentative explanations of why behaviours occur. With this understanding, the interventions target causes of changes in behaviour [30, 31]. The development of the CENBA programme was underpinned by Bandura’s self-efficacy theory [32], which had been used in a study to develop a mobile app for women treated for BC [33]. Four constructs in the self-efficacy theory include performance accomplishment, vicarious experiences, verbal persuasion, and emotional arousal. *Performance accomplishment’* refers to experiences gained when a new challenge is accomplished. *‘Vicarious experiences’* refers to the motivation gained from observing people completing a task. *‘Verbal Persuasion’* refers to the encouragement from receiving verbal feedback while undergoing a complex task. *‘Emotional arousal’* refers to the emotional responses triggered by stressful circumstances. High emotional arousal in people usually hampers performance, as fear generated by stressful situations can elevate anxiety and reduce self-efficacy. However, the likelihood of success increases if the individual’s fear can be controlled [32, 34]. “Performance accomplishment” construct was addressed by the BC education and coping skills training components of the CENBA programme. It was hypothesised that as the participants were exposed to BC education and learned coping skills, their self-efficacy in handling the demands of the chemotherapy experience would improve, thus giving them a sense of performance accomplishment. The discussion forum addressed the “vicarious experiences” construct. This component provided a means of psychological support and interaction between the participants. Also, the “verbal persuasion” and “emotional arousal” constructs were addressed by the nurse consultation component. This component allowed the nurses to respond to the participants’ concerns and provide psychological support.

#### Description of the CENBA programme

The CENBA programme contains four components: BC education, coping skills training, discussion forum, and nurse-led consultation. The first three components were delivered via the mobile app, while the consultation was conducted over the phone. The programme was delivered over six weeks. BC education was delivered asynchronously for 30 minutes per week. It covered information on BC, locally available food choices, exercise, and how to manage the side effects of chemotherapy. Coping skills training was delivered asynchronously for 30 minutes per week in weeks 3, 5 and 6, covering coping resources and strategies. A real-time discussion forum was conducted for 30 minutes every week. It was moderated by the principal investigator and featured discussion topics about the participants’ experiences. The nurse-led consultation provided informational and psychological support and was delivered through a 15-20-minute phone call in weeks 1, 3 and 5. The CENBA programme was made available to the participants free of charge. The details of the intervention are covered in the Supporting Information.

#### Content validation

The content was validated by the research team members and patients undergoing BC treatment. Comments from the team members were obtained concerning the intervention content’s accuracy, adequacy and appropriateness. Two patients undergoing BC chemotherapy were invited to comment on the intervention content to ascertain the ease of understanding.

#### Technical support

A research assistant, who was not involved in data collection, trained the intervention group for 30 minutes on how to install and use the app. Participants were given a telephone number to call in case of any technical issue that needed to be fixed during app usage. Technical support was provided from 8:00 a.m. to 5:00 p.m. daily.

### Randomisation

The randomisation scheme was generated by the website- Research Randomizer (https://www.randomizer.org/). Randomisation was performed by a research assistant trained in the procedure and was not involved in selecting the participants, delivering the intervention, or evaluating the results. Then, the research assistant prepared sequentially numbered opaque sealed envelopes with a card inside showing the group assignment. The participants assigned to the intervention group received the CENBA programme and standard care, and those assigned to the control group received standard care only.

#### Control group

In each institution, the control group received routine medical care from nurses and doctors during clinic attendance. This involved regular oncology care, instruction about chemotherapy and its side effects, and general health education. During clinic visits, nurses typically gave verbal education for about 15 minutes each day.

### Outcomes

#### Feasibility

In this study, feasibility was measured based on consent, attrition, and completion rates. The consent rate was defined as the percentage of participants who signed the consent form and agreed to participate in the study. The research assistants noted the number of participants who consented, and for those who refused to participate, their reasons for declining were documented and investigated. The attrition rate was defined as the percentage of participants who withdrew from the study. The number of withdrawals and their reasons were noted. The completion rate refers to the percentage of participants who had access to the CENBA programme and completed the intervention.

#### Acceptability

The acceptability of the intervention referred to the participants’ level of satisfaction with the intervention. This was explored through qualitative interviews conducted with the participants in the intervention group at T1. Open-ended questions were posed to the participants on their experience of using the intervention, benefits of the intervention, ease of using it, challenges encountered while using the intervention, ways the intervention could be improved, and willingness to recommend the intervention to others.

### Data collection

Data were collected at the institutions’ oncology units. The co-researchers, nurses in the oncology units, introduced the CENBA programme to eligible women. For those interested, their eligibility was confirmed, and a consent form was signed after they were given detailed information about the study. Three female research assistants with Bachelor’s degrees in nursing were trained by the principal investigator for data collection. One conducted a baseline assessment, and another, who was blinded to the allocation status of participants, collected post-intervention data after six weeks to prevent bias in the post-intervention assessment, while the third research assistant conducted individual semi-structured in-depth qualitative interviews with the intervention group post-intervention. The interviews were conducted virtually via 30-minute telephone calls using an interview guide that had been pilot-tested by the research team. Interviews continued until data saturation was reached. The recordings were conducted with participants’ consent and transcribed verbatim.

### Data analysis

Quantitative data were analysed using the Statistical Package for the Social Sciences (SPSS) version 26. The recruitment, retention and attrition rates were described as percentages. Homogeneity between the sociodemographic characteristics and outcome variables of the intervention and control groups were compared using the Mann-Whitney U test for continuous variables and the Chi-Square or Fisher’s exact test for categorical variables. Missing data were accounted for using the last observation carried forward method [35].

The analysis of the qualitative data followed a thematic approach using NVivo software [36]. First, the research team familiarised themselves with the data by reading and re-reading the transcripts. Initial codes were then generated by the principal investigator to identify significant features of the data. These codes were organised into potential themes by examining patterns and relationships. The themes were reviewed and refined to ensure they accurately represented the data, and each theme was defined and labelled to clarify its meaning. Finally, a comprehensive report was prepared to present the findings, including detailed descriptions and illustrative quotes from participants. An expert in qualitative research contributed to the writing and review process to ensure rigor This systematic approach ensured a thorough analysis of participants’ perceptions and experiences.

### Ethical considerations

Ethics approval was sought and obtained for the study from The Chinese University of Hong Kong- New Territories Eastern Cluster Clinical Research Ethics Committee (CREC no: 2021.693), LASUTH (LREC/06/10/1792) and LUTH (ADM/DCST/HREC/APP/4888). Approval to use the instruments was obtained from the original authors. The participants were given an information sheet containing detailed information about the study, after which they gave written consent by completing and signing the consent form presented by the research assistant in the presence of a witness. They were assured that participation is voluntary and that their decision to participate or otherwise will not affect their treatment. Similarly, they were assured of anonymity and confidentiality.

## Results

### Characteristics of participants

The 30 participants were between 29 and 65 years old, with a mean age of 46.67 ± 9.25 years. Most were married (93.3%) and had a tertiary education (83.3%). More than half of the participants (56.7%) were in the early stage of BC (stages 1 and 2), while a few (23.30%) were in the late stage of BC (stages 3 and 4). Around two-thirds (66.7%) often or always subscribed to the Internet and predominantly lived with their family (96.7%). The sociodemographic characteristics of the participants are summarised in Table 1.

**Table 1:** Participants’ socio-demographic characteristics (N = 30).

Characteristics	Intervention Group	Control Group	*p*
	[n = 15, *f* (%)]	[n = 15, *f* (%)]	
**Age** (Mean, SD)	48 ± 8.3	45 ± 10.1	0.43^a^
**Marital status**			0.50^b^
Single	1 (6.7)	2 (13.3)	
Married	13 (86.7)	12 (80)	
Separated	0 (0)	1 (6.7)	
Widowed	1 (6.7)	0 (0)	
**Religion**			0.76^b^
Christianity	14 (93.3)	14 (93.3)	
Islam	1 (6.7)	1 (6.7)	
**Education level**			0.51^b^
	1 (6.7)	2 (13.3)	
Secondary	1 (6.7)	0 (0)	
Tertiary	13 (86.7)	13 (86.7)	
**Stage of breast cancer**			0.80^b^
Stage 1	1 (6.7)	2 (13.3)	
Stage 2	6 (40)	8 (53.3)	
Stage 3	2 (13.3)	2 (13.3)	
Stage 4	2 (13.3)	1 (6.7)	
Don’t know	4 (26.7)	2 (13.3)	
**Frequency of internet subscription**			0.30^b^
Rarely	1 (6.7)	3 (20)	
Sometimes	2 (13.3)	4 (26.7)	
Often	6 (40)	6 (40)	
Always	6 (40)	2 (13.3)	
**Weeks since commencement of chemotherapy**			0.58^b^
1–3 weeks	2 (13.3)	5 (33.3)	
4–7 weeks	7 (46.7)	3 (20)	
8–11 weeks	3 (20)	3 (20)	
12 weeks and above	3 (20)	4 (26.7)	
**Who are you living with?**			0.80^b^
Alone	1 (6.7)	0 (0)	
Family	11 (40)	6 (40)	
Husband	3 (13.3)	5 (33.3)	
Sister	0 (0)	1 (6.7)	
Children	0 (0)	3 (20)	
**Family Income**			0.26^b^
Less than 20,000NGN (30USD)	0 (0)	2 (13.3)	
21,000–50,000 (70USD)	4 (26.7)	4 (26.7)	
51,000–100,000 (140USD)	2 (13.3)	4 (26.7)	
101,000–150,000 (210USD)	1 (6.7)	2 (13.3)	
Above 150,0000	8 (53.3)	3 (20.0)	

*Note*: ^a^ = Mann-Whitney U test; ^b^ = Fisher’s exact test

### Feasibility of the intervention

**Fig 2:**
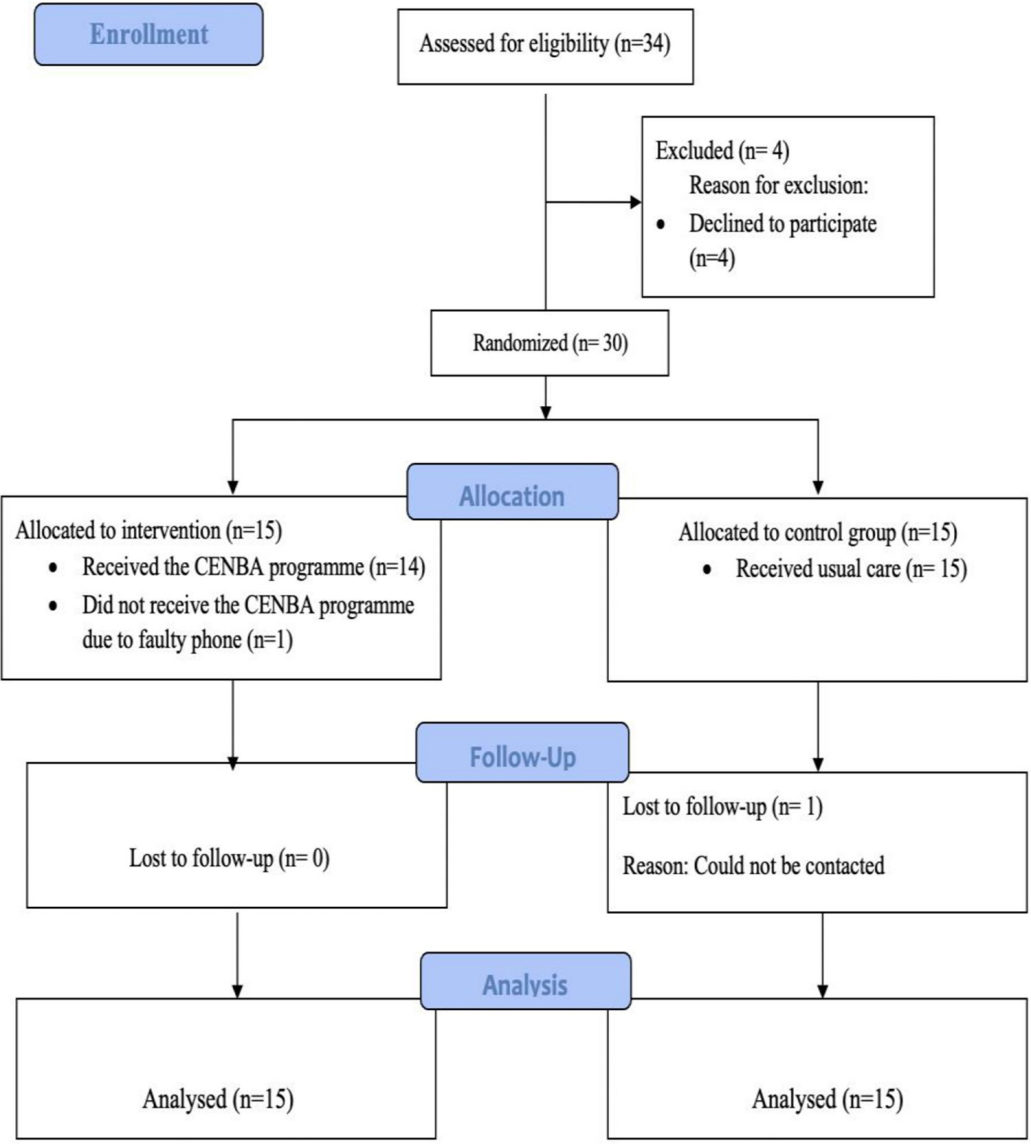
CONSORT flow diagram of participant recruitment, allocation and attrition.

Of the 34 participants approached, four declined to participate; hence, the consent rate was 86.7%. Of the 30 recruited participants, one from the intervention group did not receive the allocated intervention due to a faulty mobile phone; hence, the completion rate for the intervention group was 93.3%, and the attrition rate was 6.7%. The completion and attrition rates were the same for the control group, as one participant from the control group was lost to follow-up because she could not be contacted. Overall, the completion rate for the pilot study was 93.30%, and the attrition rate was 6.70%. Participant recruitment, allocation, and attrition are presented in Fig 2.

Data collection at baseline and post-intervention was hitch-free. The participants took, on average, 20 to 25 minutes to complete the questionnaires. The participants had no difficulty using the mobile app as the research assistants carefully explained its usage during recruitment. They had access to the app for six weeks, and during those weeks, they attended six sessions of BC education, which were released on a weekly basis. Similarly, the coping skills training was conducted in Weeks Three, Five, and Six, while the discussion sessions proceeded for 30 minutes per week. The nurse-led consultations proceeded for 15 to 20 minutes each week. They were hitch-free, as the participants were willing to discuss their concerns with the oncology nurses who were the co-investigators. The participants in the control group received standard care from the participating hospitals, which entailed a 15-minute health talk during every clinic attendance. Overall, the intervention was feasible.

### Acceptability of the intervention

Fourteen participants who completed the intervention were interviewed individually after six weeks. The interviews lasted 15 to 20 minutes. Overall, the intervention was acceptable. Two major themes emerged: the beneficial effects of the intervention and user perceptions of the app. (Table 2).

**Table 2:** Themes and subthemes from interview of participants.

Themes	Sub-themes
Beneficial effects of the intervention	Knowledge acquisition on BC and chemotherapy Opportunity for clarification of misconceptions Psychological support received with app usage
User perceptions of the app	Ease of usage Enjoyable experience with the app Recommendations for the app

### Theme 1: Beneficial effects of the intervention

All participants reported that the intervention significantly enhanced their understanding of BC and chemotherapy, addressing gaps left by traditional medical settings. It provided essential information on cancer’s causes, chemotherapy symptoms, and practical advice on diet and exercise. Additionally, it served as a valuable resource for correcting misconceptions about cancer and treatment, thus alleviating fears. The intervention also offered psychological support by connecting participants with others in similar situations, fostering a sense of community and mutual encouragement, which helped participants feel less isolated and more hopeful throughout their treatment. This is captured under the sub-themes below:

#### Knowledge acquisition on BC and chemotherapy

All participants opined that the intervention enlightened them on areas like causes of BC, symptoms of chemotherapy, diet, exercise and how to cope with the treatment. Similarly, some observed that as the hospital situation did not provide much opportunity for them to get answers to their questions, the intervention bridged the gap.

*“I mostly joined the interactive session, I was able to gain one or two things from people who are experiencing the same chemo”* (P6, 39 years, Teacher)

*“There were some things I wasn’t told about in the hospital*, *like causes*, *symptoms of chemo*, *and the effects*. *It was only on the app that I got to know the things to do*, *exercises*, *and type of food to eat; it has been very helpful”* (P2, 57 years, Teacher).

*“I was able to know how to manage reactions after chemo*. *It’s been of great help to me even while taking my chemo*, *you know*. *I never knew the reactions would be that bad or that everyone would react to the treatment differently*, *not until I used the app and went through the videos and contents*. *So*, *I was informed about the reactions and all that”* (P12, 36 years, Businesswoman).

#### Opportunity for clarification of misconceptions

Many participants observed that the intervention provided a means for them to clarify various misconceptions about BC and chemotherapy. As a result, their fears were allayed.

*“You know, I used to think that I got cancer because I sinned; it was after I joined the app that I learned this is not true. From my interaction with others on the app, I learned that this is what other people experience too. It was beneficial”* (P11, 40 years, Civil Servant).

*“…you know ehm*, *as they say*, *it is not really good to believe in what people say*. *While I was going for the chemo*, *they would tell us*, *don’t eat this*, *don’t eat that*, *but from the app and the teaching*, *I realised that we could eat certain things in moderation*. *I restricted myself from eating many things because they asked us not to eat the things I felt like eating*. *At a time*, *I was just taking water*. *But anyway sha*, *as they say*, *we learn every day; I’m happy I was involved with the forum”* (P4, 44 years, Photographer).

#### Psychological support received with app usage

The participants declared that the app afforded them the opportunity to discuss with patients who were having similar experiences. They found this to be very supportive.

*“…I was able to share my experiences with other persons. They shared their own experience too and we were able to support each other. This was very helpful”* (P3, 45 years, Insurance Broker).

*“You know*, *during the interactive session*, *you hear people talk; you get to know you are not in it alone*. *With these words*, *we get really encouraged and hopeful that we will get better”* (P6, 39 years, Teacher).

### Theme 2: User perceptions of the app

Participants generally perceived the intervention positively, noting that the app was easy to use and navigate, with unanimous agreement on its simplicity. They appreciated the straightforward design, which facilitated smooth access to various features, including discussion and educational forums. The enjoyable aspects of the app were highlighted, particularly the interactive sessions and educational content, which participants found engaging and supportive. Many valued the app’s interactive nature and the moderator’s patient approach. However, some participants suggested improvements, such as enhancing the interaction quality and addressing issues related to data access and reminders for forum sessions. These insights reflect both the app’s strengths and areas for potential enhancement.

#### Ease of usage

Participants unanimously commented on the app’s ease of use, agreeing that it was user-friendly.

*“I found the app very easy to use. The lady who introduced the app to me took the time to explain how to use the app. She logged me into the app. So anytime I want to use the app, I just click on it. It was easy to navigate from the discussion forum to the education forum and view the pictures and videos”* (P2, 57 years, Teacher).

*“I found the intervention straightforward*, *and ehn it is so simple enough” …”* (P11, 40 years, Civil Servant)

*“The app did not pose any challenge and is not complicated*. *Also*, *I made sure I had enough data*, *so I’m always on the internet…”* (P4, 44 years, Photographer).

#### Enjoyable experience with the app

This subtheme reflects the participants’ views on app features. Many opined that they enjoyed the app’s educational and interactive features.

*“I really enjoyed the forum. Getting to interact with one another was very helpful. The way the nurses interacted with us was encouraging. Also, I enjoyed the educational aspect”* (P14, 51 years, Civil Servant).

*“I enjoyed the interactive session most*. *I like the way the moderator carries everybody along*. *During the interactive session*, *there was nothing like bullying*. *The moderator was so gentle with us*, *irrespective of the background*. *Ehn*, *there was nothing like shutting people down*. *If it were to be others*, *they wouldn’t even allow you to talk*. *So*, *the moderator was very patient; I like that”* (P4, 44 years, Photographer).

#### Recommendations for the app

Some participants gave some recommendations on how to improve the app. Their suggestions are captured in the statements below:

*“I feel the interaction on the app can be better. I feel the responses are not enough. I also think some people might not have data and due to that, they might not be able to participate, so it will be good if you can help with that”* (P1, 45 years, Teacher).

*“…at times*, *I forget*. *It is just the very day I will remember I have to attend the discussion forum in the evening ooo*, *but ehn*, *it will have been after 8 or 9 pm*. *If you can send us a text message a few minutes before we start the forum*, *it will be helpful”* (P2, 57 years, Teacher).

## Discussion

This study has documented the development of the CENBA programme, a mHealth intervention for providing psychoeducational support for Nigerian women diagnosed with BC undergoing chemotherapy. To the best of our knowledge, this intervention is the first nurse-led mHealth PEI for this population in Nigeria. Nigeria’s mobile market is the fastest growing in Africa, with 187.9 million mobile connections as of January 2021, and mobile phone usage increased during the COVID-19 pandemic [37, 38]. Although mobile phone usage is increasing, little is known about the effect of a mHealth intervention on the psychological issues of Nigerian women diagnosed with BC receiving chemotherapy.

Findings suggest that using the mobile app among this population is feasible. It was not difficult to recruit the participants. Also, the attrition rate was not high. Findings suggest that the intervention was acceptable. The participants did not experience any difficulty in using the app. Similarly, all the participants unanimously agreed that they found the intervention helpful and were willing to recommend it to women in the same situation. This was similar to the findings of two studies conducted in Iran and Taiwan, where a mobile app was developed to support women diagnosed with BC, and the app was reported to be feasible and acceptable among the population [39–41].

Some factors might have influenced the acceptability of the intervention. First, a high level of education was reported by Tola et al. [42], in which 75% of the participants had a tertiary level of education. According to a survey by the National Demographic Health Survey in 2019, at least half of the women in Nigeria had completed secondary school [43]. Statistics also showed that the greatest proportion of women with secondary education or higher (68%) was found in Lagos State, where this study was conducted [44]. Also, two-thirds of the participants often or always subscribe to the Internet. In 2027, 117 million Nigerians are expected to use the Internet, according to the projection [45]. Another factor might be the app design and the language of instruction, which were simple, and the words were large enough to be seen. This aligned with the position of Luna et al. [46], who noted that mobile apps must be simple to operate, the user interface must be clear, and the colour must be well contrasted. Lastly, the technical support provided to the participants during the intervention might have contributed to high user engagement. The participants were given a number to call if they had technical difficulties with the app. The need for technical support was highlighted in a previous app-based study [47], and thus, it should be provided in future related studies.

It is worth noting that some of the participants did not access the educational content and discussion components of the CENBA programme often enough when their symptoms were unbearable, as they only consulted the oncology nurses during this period. This was similar to the poor usage of the mobile app for Chinese women with BC receiving chemotherapy when they felt too sick as reported by Zhu et al. [33]. This also aligned with previous studies that reported the association between poor health status and infrequent usage of digital devices [48, 49]. Another factor is culture. The intervention included locally available nutritious diets and coping resources available in the Nigerian context. Other alternatives that were not covered in the BC education component were also raised by the women during the discussion sessions. This was also an avenue for clarifying various misconceptions regarding diet. Dietary concerns were also covered in previous mHealth interventions for women with BC receiving chemotherapy [33, 39, 50].

Similarly, the BC education component in the CENBA programme provided information on resources available in the community for women with BC, such as religious organizations for Christians and Muslims, as these were the dominant religions among the participants. Furthermore, information was provided on the need to balance orthodox medicine and spirituality, as this was stressed by the participants in the study in Phase II.

The study participants gave some recommendations. Some participants observed insufficient interaction among the participants during the discussion sessions. Two reasons were suggested: inadequate internet subscription bundles and the need for text message reminders for them to join the interaction. They recommended that data subscription should be provided for subsequent trials. Although statistics from the National Bureau of Statistics in Nigeria revealed that Lagos, where this study was conducted, had the highest internet subscription as of March 2022 [51], we found that many participants complained that the treatment was financially draining, which might influence their frequency of internet subscription. It is important to note that Nigeria is one of the countries with the most extreme poverty [52], and financial toxicity has been reported among Nigerian women undergoing BC treatment. In a previous study, the out-of-pocket cost of BC was high, as 72% of the households had to borrow money to support their treatment [53]. As of the time of this study, 53.4% had a monthly household income of 30–140 USD. Furthermore, evidence suggests that the treatment plan of BC patients receiving chemotherapy in Nigeria is hampered due to a paucity of funds, as chemotherapy is an out-of-pocket expense, and many patients have to borrow money to survive. Knapp et al. [53] reported that over 90% of households with Nigerian women undergoing BC treatment in their study had experienced catastrophic health expenditures. The high cost of chemotherapy in this setting also affected the availability of funds for internet subscriptions, which was the reason for providing stipends (3 USD) to support internet access for the participants in this study. Although a previous study had suggested that some Nigerian women receiving BC chemotherapy reported that they subscribed to the Internet frequently [26], this study found that 13% of the respondents rarely subscribed to the Internet, and 20% sometimes subscribed to the Internet. Hence, we recommend that internet subscriptions for patients should be considered for subsequent studies. Similarly, we recommend that reminders in the form of text messages might be considered for subsequent trials to improve the interaction on the app.

The various components of the CENBA programme, such as BC education, discussion forum, psychological support, and nurse consultation, were similar to the components covered by previous interventions designed for this population. In previous studies, these components improved emotional functioning, self-efficacy, self-esteem, and reduced symptom prevalence, symptom interference, symptom distress and supportive care needs [25]. As the process of developing the mHealth PEI has been documented, evidence from this study can guide the design of similar interventions in future studies. Similarly, as nurses’ role in advancing Nigeria’s health system has been reported [54] and Nigerian nurses have been involved in successfully designing digital interventions [55], we recommend the design of future mHealth interventions to meet the psychoeducational needs of this population. With the usage of mHealth psychoeducational interventions, it is hoped that Nigerian women with BC will receive adequate psychological care while receiving chemotherapy.

It is important to consider some of the limitations of this study in interpreting the findings. First, as the study was conducted in Lagos, Nigeria, which is one of the states in the southwestern part of Nigeria, and convenience sampling was utilised, it did not reflect the experiences of patients with BC in other states in the southwest and other geopolitical zones in Nigeria. Although the study recruited participants from various tribes in Nigeria, as it was conducted in Lagos, which is home to people from various Nigerian tribes, it is worth noting that the study participants were urban dwellers. Thus the study results are not generalisable to rural Nigerian dwellers. Similarly, as the study only recruited those who could speak English, this might limit the generalisation to Nigerian women who are not proficient in English. Hence, it is recommended that future research consider translating the intervention to other Nigerian languages.

Additionally, since the intervention was culturally tailored for Nigerians, evidence from this study is not generalisable to citizens of other countries. Furthermore, while a small sample size is acceptable for a pilot study, the intervention’s effectiveness on this population’s psychological outcomes is not yet established. A trial with a large sample size is indicated. Similarly, we did not initially distinguish between patients receiving neoadjuvant and adjuvant chemotherapy. This lack of differentiation might impact the generalizability of our findings, as the therapeutic goals and patient experiences can vary between these treatment modalities. Future studies should consider stratifying patients based on their specific chemotherapy phase. Also, while using the Last Observation Carried Forward method to handle missing data helped to maintain our sample size and facilitate data analysis, it carries the risk of introducing bias into the study. Future studies might conduct additional analyses to account for such potential bias. Also, as the usage of mHealth in this population in Nigeria is relatively new and has very limited evidence, this study has provided evidence for integrating mHealth into BC chemotherapy.

## Conclusion

This feasibility study presented the process of developing the CENBA programme aimed at providing psychoeducational support for Nigerian women diagnosed with BC undergoing chemotherapy. The programme’s development was informed by evidence from a systematic review, a qualitative study involving Nigerian women with BC, and Bandura’s theory of self-efficacy. The programme was found to be acceptable and feasible for this target population. Nevertheless, further refinement based on participant feedback and additional testing with a larger sample size is recommended to evaluate its effectiveness.

## Supporting information

S1 Table Details of the CENBA programme- a mHealth psychoeducational intervention. (DOCX)

S2 Table CONSORT 2010 checklist of information to include when reporting a randomised trial. (DOC)

S1 File Study protocol. (DOCX)

S2 File Copyright permission. (PDF)

S3 File Inclusivity in global health questionnaire. (DOCX)

S4 File Study data. (CSV)
