# Supplementary material for: Development and feasibility of an mHealth intervention for psychoeducational support of Nigerian women diagnosed with breast cancer undergoing chemotherapy: A pilot randomized controlled trial
Source: PLoS One. 2024 Nov 25;19(11):e0314365. doi: 10.1371/journal.pone.0314365 (PMC11588262; doi:10.1371/journal.pone.0314365)
Supplement: S1 Table — (DOCX) [file pone.0314365.s001.docx]

| **S1 Table. Details of the CENBA Programme- a mHealth Psychoeducational Intervention**  **Session** | **Week** | **Discussion Forum** | **Education forum** | **Mode of Delivery** | **Activity** | **Components** |
| --- | --- | --- | --- | --- | --- | --- |
| Session 1 | 1^st^ week | - Self and group introduction. | - Answer questions and clarification misconceptions - Allay concerns and clarify misconceptions about chemotherapy. | - Recorded version for 30 mins BC education, 15 mins video from BC survivor, and 30mins real-time discussion via chat. | - - Provide a relaxing atmosphere that will facilitate the sharing of ideas. - - Identify myths and misconceptions on BC. - - Correct misconceptions. - - Provide accurate knowledge on the topics. - - Deliver a 15-20 minutes personal consultation for participants via phone call. | BC education, discussion forum, psychological support, nurse-led consultation |
|  |  | - How did you feel when you were diagnosed with BC? |  |  |  |  |
|  |  | - Definition of BC |  |  |  |  |
|  |  | - Causes of BC |  |  |  |  |
|  |  | - What are the side effects you have experienced so far during your chemotherapy? |  |  |  |  |
|  |  | - What are the psychological issues you have experienced so far? |  |  |  |  |
| Session 2 | 2^nd^ week | - What are side effects of chemotherapy you have experienced so far? - How have you been managing the side effects? | Managing nausea and vomiting, fatigue, disfigurement, nutritional deficiencies, and other identified side effects of chemotherapy. | - Recorded version for 30mins BC education and 30mins real time discussion via chat | - - Provide a relaxing atmosphere that will facilitate the sharing of ideas. - - Provide accurate knowledge on the topics. - - Engage participants on a personal consultation as the occasion demands. | BC education, discussion forum, coping skills training, psychological support, and nurse-led consultation |
| Session 3 | 3^rd^ week | - Diet and exercise during chemotherapy | Teaching participants the right diet during chemotherapy and different forms of exercises that they can tolerate. | - Recorded version of BC education (30 mins). - Coping skills training and real-time discussion via chat (30 mins). | - - Provide a relaxing atmosphere that will facilitate the sharing of ideas. - - Provide accurate knowledge on the topics. - - Deliver a 15-20 minutes personal consultation for participants via phone call | BC education, discussion forum, coping skills training, psychological support, and nurse-led consultation |
| Session 4 | 4^th^ Week | Sharing experiences and learning from others | Facilitating sharing success stories and challenging moments | 30 mins real time discussion via chat | - - Provide a relaxing atmosphere that will facilitate the sharing of ideas. - Provide accurate knowledge on the topics. - - Engage participants on a personal consultation as the occasion demands. | Discussion forum, psychological support, nurse-led consultation |
| Session 5 | 5^th^ Week | - Coping through spirituality. - How has religion and spirituality influenced your chemotherapy experience? | Sharing strategies for coping through spirituality. | - Recorded version for coping skills training, - Real-time discussion via chat (30 mins). | - - Provide a relaxing atmosphere that will facilitate the sharing of ideas. - - Provide accurate knowledge on the topics. - - Deliver a 15-20 minutes personal consultation for participants via phone call | BC education, discussion forum, coping skills training, psychological support, and nurse-led consultation |
| Session 6 | 6^th^ week | What are the coping strategies you have used so far through your chemotherapy journey? | - Reinforcing positive coping strategies identified including - Teaching participants how to utilize positive coping strategies - Teaching participants how to avoid negative coping strategies | Recorded version for coping skills training and 30mins real time discussion via chat | - - Provide a relaxing atmosphere that will facilitate the sharing of ideas. - - Provide accurate knowledge on the topics. - - Engage participants on a personal consultation as the occasion demands | BC education, discussion forum, coping skills training, psychological support, and nurse-led consultation |
