# Supplementary material for: Development and feasibility of an mHealth intervention for psychoeducational support of Nigerian women diagnosed with breast cancer undergoing chemotherapy: A pilot randomized controlled trial
Source: PLoS One. 2024 Nov 25;19(11):e0314365. doi: 10.1371/journal.pone.0314365 (PMC11588262; doi:10.1371/journal.pone.0314365)
Supplement: S1 File — (DOCX) [file pone.0314365.s003.docx]

**Effect of a mHealth psycho-educational intervention (mPEI) on self-efficacy, coping, and psychological distress among women receiving chemotherapy for breast cancer in Nigeria: A randomised controlled trial**

**A Research Proposal**

**Submitted to**

**The Joint Chinese University of Hong Kong – New Territories East Cluster Clinical Research Ethics Committee**

**By**

**Akingbade Oluwadamilare**

**1155150005**

**Professor Ka Ming Chow**

**Supervisor**

**December 2021**

# Table of Contents

**Title page …………………………………………………………………………………….1**

**Table of Content………………………………………………………………………………2**

**Introduction……………………………………………………………………………… …3-5**

**Aims and Objectives………………………………………………………………………….5**

**Hypothesis…………………………………………………………………………………....6**

**Development of the Intervention………………………….…………………...…………….6-8**

**Design of the mPEI programme……………………………………………………………8-13**

**Components and contents of the Intervention……………………………...8-12**

**Content validation………………………………………………………........12**

**Intervention format………………………………………………………...….12**

**Intervention duration……………………………………………………….…12**

**Intervention dosage…………………………………………………………...13**

**Intervention delivery…………………………………………………………13**

**Technical support…………………………………………………………….13**

**Piloting of the Study……………………………………………………………………...14-18**

**Aim and Objectives…………………………………………………………...14**

**Design………………………………………………………………………...14**

**Sample size planning…………………………………………………….......14**

**Setting………………………………………………………………………...14**

**Inclusion criteria……………………………………………………………...14**

**Exclusion criteria………………………………………………………….….15**

**Research assistant training…………………………………………………...15**

**Outcome measures………………………………………………………...15-17**

**Data collection……………………………………………………………17-18**

**Data analysis…………………………………………………………………18**

**Main RCT………………………………………………………………………...19-23**

**Study design………………………………………………………………….19**

**Study setting………………………………………………………………….19**

**Sample size planning…………………………………………………………19**

**Outcome measures…………………………………………………………...20**

**Fidelity of the intervention…………………………………………………...20**

**Data collection procedure………………………………………………...20-21**

**Participant recruitment……………………………………………....20**

**Blinding…………………………………………………………...….21**

**Randomisation……………………………………………………………….21**

**Process evaluation………………………………………………………...21-22**

**Data analysis…………………………………………………………………22**

**Ethical considerations…………………………………………………….22-23**

**Impact of the study on Nursing Practice……………………………………..24**

**References………………………………………………………………………………...25-36**

# Introduction

One in eight cancers diagnosed worldwide in 2020 were breast cancers (BCs); likewise, one in four of all new cancer cases in women are BCs (Global Cancer Observatory [GLOBOCAN], 2020). Globally, female BC surpassed lung cancer in 2020 to become the most commonly diagnosed cancer, affecting 2.1 million women each year with new cases expected to increase by 46.5% in the year 2040 (GLOBOCAN 2020; World Health Organization [WHO], 2019). In African countries, BC is the most frequent cause of cancer mortality causing one in five deaths in African women (Vanderpuye et al., 2017). In Nigeria, BC was the most common cancer recorded in the year 2018, accounting for 22.7% of all cancer cases and responsible for 16.4% of all cancer deaths (WHO, 2020). While the mortality from BC in a developed country like America is 19%, that of Nigeria is triple (Federal Ministry of Health, 2018).

Several treatment modalities exist for BC, which include chemotherapy, targeted therapy, surgery, and hormone therapy (Diaby et al., 2015; Matsen & Neumayer, 2013). Also, various side effects have been found to be specific for the various treatment modalities which make the BC treatment highly challenging. Focusing on women receiving BC chemotherapy is important as the usage of chemotherapy in treating women with BC is universal (Fan et al., 2014; Gold et al., 2014), cutting across all stages of BC (Du & Goodwin, 2001), and used before and after surgery (Charfare et al., 2005). As various side effects have been reported for the various treatment modalities (Lo et al., 2017); also, symptom clusters of pain, fatigue, anxiety, and depression have been documented among women receiving BC chemotherapy (Browall et al., 2017; So et al.,2009).

It has been estimated that 30-50% of women diagnosed with BC have psychological concerns somewhere along their illness trajectory (Gupta et al., 2020; Hewitt et al., 2004). Psychological concerns have been reported as one of the most common adverse effects experienced by BC women receiving chemotherapy (Henderson et al., 2012). Common psychological concerns reported include symptom burden, symptom distress, fear, anxiety, depression, deteriorated quality of life, poor social support, and unmet supportive care needs (Au et al., 2012; Hewitt et al., 2004; Browall et al., 2017). Furthermore, self-efficacy in coping, which refers to an individual’s belief in her capacity to cope effectively with or adjust to a particular situation (Bandura, 1977; Chesney et al., 2006), has been reported to reduce significantly in this population (Akin et al., 2008). Furthermore, evidence has shown that assessing self-efficacy in coping in cancer patients could facilitate identifying coping strengths that need to be reinforced or coping weaknesses that need to be modified through appropriate interventions with a bid to minimising the psychological burden of cancer (Heitzmann et al., 2001). Enhancing coping with chemotherapy among BC patients is important as this can help improve emotional state of these women and encourage them to adhere to the treatment (Watkins et al., 2017).

Although psychological concerns are common in this population; they are not inevitable (Guo et al., 2013), as appropriate interventions can be targeted towards mitigating the psychological concerns. Similarly, the transactional model of stress and coping has demonstrated that after a stressful condition, an individual would assess his/her available coping resources and adopt coping strategies to cope with the situation (Glanz et al., 2002).

To address psychological concerns of women diagnosed with BC, some interventions have been suggested. Guarino et al. (2020) conducted a systematic review on interventions for addressing psychological concerns of women diagnosed with BC and identified three major categories of such kind of interventions, including cognitive-behavioural interventions, psychoeducational interventions (PEI), and supportive-expressive interventions (SEIs). A medium effect size was found for PEIs and SEIs on improving anxiety, depression, mood, and quality of life. As educational needs have also been reported in this population, PEIs are important in addressing the psychological and educational concerns (Au et al., 2012; Fanakidou et al., 2017). PEIs have been defined as a broad range of activities that combine structured transfer of knowledge through education with psychological activities like counselling and supportive interventions (Oncology Nursing Society, 2021).

While the need to provide PEIs for BC women has been documented in literature; mHealth, defined as “health-related services delivered through mobile communication devices” (Whitaker 2012) has been suggested as a medium to deliver these interventions because mobile phones have become ubiquitous (Mobasheri et al., 2014). Similarly, Okunade and colleagues (2020) also noted that one of the developments after the COVID-19 pandemic in Nigeria would be the integration of telemedicine into the care of oncology patients; however, evidence to guide this integration is still being gathered and not yet established.

Although the benefits of mHealth interventions have been reported along the BC illness trajectory (Beauchamp et al., 2020; Galiano-Castillo et al., 2017; Jongerius et al., 2019), mHealth interventions used specifically in women receiving BC chemotherapy is unclear and the effect of these interventions on psychological concerns in this population is inconsistent. Similarly, as the usage of mHealth in this population is relatively new and with very limited evidence, this study, which to the best of our knowledge is the first in Nigeria, will generate evidence for integrating mHealth into care for women with BC receiving chemotherapy. Similarly, nurses will be informed of the effectiveness of a mHealth psychoeducational intervention (mPEI) for this population. It is hoped that the psychological and educational concerns of Nigerian women with BC will be adequately addressed while receiving chemotherapy.

**Aim and Objectives**

The study aims to develop and implement a culturally adapted mPEI for BC women receiving chemotherapy in Nigeria. The objectives of the study are to: 1) evaluate the effectiveness of the programme on self-efficacy (primary outcome), coping, symptom distress, anxiety, depression, and quality of life (secondary outcomes) among women with BC receiving chemotherapy; and 2) explore the participants’ experiences and feelings towards the intervention.

**Hypothesis**

It is hypothesised that participants who receive the mPEI, when compared with the control group at baseline and immediately after the intervention, will demonstrate significantly higher level of self-efficacy, coping, quality of life, and significantly lower level of symptom distress, anxiety and depression.

**Development of the Intervention**

The development of the mPEI is guided by evidence from the systematic review, a qualitative study conducted by the research team and a theoretical framework.

A systematic review was conducted by the research team featuring seven mHealth interventions used among 799 women with BC undergoing chemotherapy. The study showed that most of the interventions were application-based and focused on psychoeducation. The major components of the interventions were education, group interaction, self-reporting of symptoms and expert consultation, and most of the intervention providers were nurses. The meta-analysis results indicated a significant improvement in the quality of life (standardised mean difference [SMD] = 0.32, 95% confidence interval [CI] [0.07, 0.58], p = .01, I2 = 17%). No significant effects were found for anxiety (SMD = 0.00, 95% CI [-0.32, 0.32], p = 1.00, I^2^ = 64%] and depression (SMD = 0.02, 95% CI [-0.17, 0.20], p = .87, I^2^ = 0%). Similarly, results from previous studies also found conflicting results. However, two of the studies included in this systematic review (Hou et al.,2020; Zhu et al 2018) suggest that further rigorous trials should be conducted to confirm the effect of mHealth interventions on these outcomes, hence, both outcomes will be measured in this study.

Individual studies suggest reduced symptom prevalence (p = .033, d =.27), symptom distress (p = .004, d = .004), symptom interference (p = .02, d = .51), supportive care needs (p < .05, d = 2.43); improved self-efficacy (p = .03, d = 0.53) and emotional functioning (p = .008, d = .30). The methodological quality of the included studies ranged from low to moderate. The study concluded that mHealth interventions might be helpful in addressing certain psychological issues experienced by women receiving chemotherapy for BC, although the evidence is still being gathered and not yet conclusive.

In order to identify the psychological and educational needs of Nigerian women receiving chemotherapy so as to develop culturally-adapted interventions, as well as to identify the perceptions on this kind of intervention, a qualitative study was conducted by the research team. A total of 32 women who had completed BC chemotherapy were recruited from two tertiary hospitals in Nigeria- Lagos State University Teaching Hospital and Lagos University Teaching Hospital. Focus group interviews were conducted and is was found that the participants perceived the proposed intervention to be feasible. The participants suggested contents for the proposed mHealth intervention. The contents include information on chemotherapy, how to deal with fear of chemotherapy, nutritious diet locally available and information on exercise. Many of the participants found the first cycle of chemotherapy extremely challenging. Psychological disturbances experienced include sadness, fear of chemotherapy, confusion, insomnia. Their needs were majorly psychological and informational. Coping resources available for the participants were support from their families, health professionals, BC survivors and their affiliated churches. Coping strategies adopted include being positive, prayer, online information, and exercise.

Regarding the theoretical framework underpinning the intervention, the self-efficacy theory was adopted to guide its development. Evidence suggests that people’s belief in their ability to handle or cope with a threat can lower their anxiety and depression as an association has been found between low self-efficacy and stress, anxiety and depression (Bandura 1995; Zulkosky, 2009). Similary, Borjalilu et al. (2017) identified an association between high self-efficacy in coping and improved quality of life among women with BC. They also reported that women with BC who had lower self-efficacy had more physical symptoms including nausea, pain, numbness, weakness, swollen hands, legs and feet.

Four constructs from the self-efficacy theory underpin this intervention. They include performance accomplishment, vicarious experiences, verbal persuasion, and emotional arousal. *‘Vicarious experiences’* refers to the motivation gained on observing people successfully completing a task (Bandura, 1997). *‘Verbal Persuasion’* refers to the encouragement gotten from receiving a verbal feedback while undergoing a complex task (Bandura 1997). *‘Emotional arousal’* refers to the emotional responses that are triggered by stressful circumstances. High emotional arousal in people usually hamper performance as fear generated by stressful situations can culminate in elevated levels of anxiety which reduces self-efficacy (Bandura, 1977). However, if the fear of the individual can be controlled, the likelihood of success is increased (Wallace & Kernozek, 2017). ‘*Performance accomplishment’* refers to experiences gained when a new challenge is accomplished successfully. Occasional failures are overcome easily after repeated success. With the implementation of the mPEI programme, self-efficacy in coping with demands of chemotherapy will be increased. With enhanced self-efficacy, there will be a reduction in anxiety, depression and symptom distress which will culminate in an improved quality of life. Evidence suggests that with the usage of a mPEI among women with BC, psychological benefits such as reduced anxiety, depression, symptom distress and improved quality of life are obtained (Fjell et al., 2020; Ghanbari et al., 2021; Lepore et al., 2014; Zhu et al., 2018).

A conceptual framework showing the relationship between the mHealth programme components and the outcomes is presented in Appendix A.

**Design of the mPEI programme**

A mobile application will be used to deliver the mPEI programme. The formulation of the app was developed by *Deltree Technologies,* an Information and Communication Technology (ICT) company based in Nigeria. Based on the self-efficacy theory, findings from the systematic review, and consultation with experts in BC management and psychoeducation, the mPEI programme contains five components: BC education, coping skills training, discussion forum, nurse-led consultation, and psychological support. The design of the intervention is presented in Appendix B.

***Components and contents***

- *BC education*

As found in studies in the systematic review (Hou et al., 2020; Zhu et al., 2018), the education component provides information on BC, chemotherapy side effects, and how to cope with chemotherapy. The education component is also guided by findings from an extensive literature review, consultation with professionals in oncology, findings from the phase II qualitative study, knowledge gained from a training the principal investigator received on psychoeducation, and based on the clinical practice guidelines developed by the National Cancer Control Initiative (National Breast Cancer, 2003). BC education will feature through the six weeks of the intervention.

BC education in the mPEI programme addresses the ‘performance accomplishment’ construct in the self-efficacy theory. As the participants are exposed to BC education and they successfully implement the lessons learnt; this will bring a feeling of performance accomplishment which will improve their self-efficacy to cope with the demands of the chemotherapy experience.

The BC education is delivered on the app in three formats namely, text with pictures, audio and video format. The language will be in English which is the official language in Nigeria. The participants will be expected to read and/or watch the BC education content within the week of posting.

As the previous qualitative study suggested, stories from BC survivors are incorporated into the educational platform in a video format for 15 minutes per week for the first, third and fifth weeks.

- *Coping skills training*

This component provides training on coping skills as evidence has shown that improving knowledge about coping resources, strategies and skills can culminate in positive appraisal while improving self-efficacy in coping with treatment (Bandura, 1977; Swanson et al., 1999; Watkins et al., 2017). There are three sessions of coping skills training. This is to cover three of the six weeks. The sessions focus on coping with chemotherapy side effects, maximising coping resources, dealing with psychological issues through religion and spirituality, maximising coping strategies and leveraging on family support.

This component addresses the ‘performance accomplishment’ construct of the self-efficacy theory. As the participants are taught coping skills, and they successfully implement these skills; this will bring a feeling of ‘performance accomplishment’, which will improve their self-efficacy to cope with the demands of the chemotherapy experience. Similarly, as the participants learn coping skills based on positive re-interpretation, acceptance, and spirituality, a positive emotion will be induced which will improve their coping with the chemotherapy experience. The participants will be expected to read and/or watch the coping skills training content within the week of posting.

- *Discussion forum*

As found in the systematic review (Hou et al., 2020; Lepore et al., 2014; Zhu et al., 2018), the discussion forum will feature discussion topics from the education, and this will be divided into sessions of six weeks and moderated by the researcher. Discussion forum in the mPEI programme will address the ‘vicarious experiences’ construct in the self-efficacy theory. As the women interact on the platform and share their success stories in adjusting and coping with the chemotherapy experience, this will encourage others and they will be motivated to complete the task which is successfully completing the chemotherapy.

The discussion forum will be implemented real time and non-real time. The real-time discussion will be at a time agreed on by the participants. Those that are not online at that time can leave further comments on various issues raised at their convenient time. The participants will be free to use a name they are comfortable with so their identity can be confidential. Similarly, they will not be required to display their pictures to maintain privacy. There will be six discussion groups in all with each group containing an average of 10 members as evidence suggests that this number allows for meaningful interaction and group administration (Biggs et al., 2020). The discussion forum will feature through the six weeks of the intervention.

- *Nurse-led consultation*

This will provide a means of answering questions and supporting participants in terms of their informational and psychological needs. This function will be provided by the oncology nurse designated for each participant through a 15-20-minute call in the first week, third week and fifth week.

This component will address the ‘verbal persuasion’ construct of the self-efficacy theory. As the participants consult the nurses, they will receive verbal encouraging feedbacks that will support them through the chemotherapy experience.

- *Psychological Support*

The psychological challenge experienced by women receiving breast cancer chemotherapy varies depending on her temperament, her other burdens, and psychological history (Greenberg, 2010). Psychological support will be provided by actively listening to the participants, provision of a means to verbalize worries and concerns, and providing informational support. The psychological support will be given on an individual basis through the nurse-consultation function. This will entail 15-20 minutes call first week, third week and fifth week by oncology nurses from the research team.

This component will address the ‘emotional arousal’ construct of the self-efficacy theory. The fear of cancer and the chemotherapy experience will be controlled through the psychological support. As fear is controlled, anxiety will be reduced and self-efficacy to cope with the chemotherapy experience will increase.

***Content Validation***

The content has been validated by an expert panel and BC patients. The expert panel comprises two oncology nurses, with at least five years’ experience in oncology nursing, an oncologist, a psychologist, a mental health nurse and a research professor. Comments from the experts were obtained with respect to accuracy, adequacy and appropriateness of the intervention content. Two patients undergoing BC chemotherapy will be invited to comment on the content of the intervention to ascertain that the contents of the intervention will be easily understood by the patients.

***Intervention format***

PEIs can be delivered through various formats, including individual, group, home, hospital, or community-based (Moore et al., 2021; Sin & Norman, 2013). For this study, as evidence from the systematic review conducted suggested that PEIs delivered through the mobile phone might be beneficial in resolving certain psychological issues in this population; the intervention will be delivered through a mobile phone. Similarly, as the intervention is multi-component, a combination of individual and group formats will be employed. The BC education, coping skills training and discussion components will be delivered on a group basis; psychological support and nurse-led consultation will be delivered on an individual basis. Furthermore, the intervention will be community-based as chemotherapy in Nigeria is delivered on an out-patient basis.

***Intervention duration***

A duration of six weeks will be considered as evidence from a previous study suggest that online PEI delivered for six weeks might be effective in improving psychological outcomes (Lepore et al., 2014).

***Intervention dosage***

The dosage which include amount, frequency, and duration of the intervention (Manojlovich & Sidani, 2008; Reed et al., 2007) will depend on the component of the intervention concerned. BC education and discussion will each be delivered for 30 minutes per week as this was an average duration and frequency found among previous studies in the systematic review earlier conducted. Stories from BC survivours will be delivered 15 minutes per week on the first, third and fifth weeks. Coping skills training will be incorporated into the BC education that will be delivered for a period of three weeks. Amount, frequency and duration of psychological support and nurse consultation will be 15-20 minutes call first week, third week and fifth week by oncology nurses from the research team.

***Intervention delivery***

To ascertain consistency of the intervention, all sessions of the BC education and coping skills training will be delivered by the principal investigator. Similarly, all discussion sessions will be moderated by the principal investigator. The BC education session will be held every Monday for 30 minutes which will be followed by a discussion forum for 30 minutes.

***Technical Support***

The research assistants will train the intervention group for 30minutes on how to install and use the app. Participants will be given the telephone number of the developer so they can call in case of any technical issue that needs to be fixed during the application usage. They can also drop a text message, or a voice note for the developer. Technical assistance will be provided 8:00am to 5:00pm from Monday to Friday.

**Piloting of the Intervention**

***Aim and Objectives***

The pilot study is aimed at testing the acceptability and feasibility of the mPEI programme for addressing psychological issues of Nigerian women undergoing BC chemotherapy and identifying participants’ comments on the programme.

The specific objectives are to: (i) test the acceptability and feasibility of using the mPEI programme and study procedures; (ii) evaluate the recruitment capability; (iii) explore participants’ comments on the intervention; and (iv) utilise the findings for amendment of the intervention for the main study.

***Design***

An assessor-blinded, two-arm RCT using a parallel group will be adopted. Qualitative data will also be collected from an online focus group discussion to assess the acceptability, perceived usefulness, and ease of usage of the intervention.

***Sample Size Planning***

According to Kato (2020), a sample size of 15-20 per group is appropriate for ensuring scientific validity of a smartphone-application based pilot study; hence, for this study, 15 participants will be utilised for intervention and control groups, respectively.

***Setting***

The pilot study will be conducted in Lagos University Teaching Hospital which is one of the centres used for the qualitative study described earlier.

***Inclusion criteria***

These will include females who: (1) are between age 18-70 (2) are newly diagnosed with BC within the recent three months; (3) currently receiving chemotherapy; (4) have access to a smartphone and internet; (5) are able to read and write in English; (6) are cognitively capable of completing the questionnaires; and (7) consent to join the study.

***Exclusion criteria***

These will be those women with a concurrent physical or mental illness (as these might act as a confounder); cognitively impaired; are older than 70 years of age as aging barriers have been noted in mhealth usage with older adults (Wildenbos et al., 2018).

***Research assistant training***

Two research assistants (RAs) that were previously trained in qualitative data collection will be further trained for one hour on subject recruitment and quantitative data collection using the data collection instruments. Their ability to properly administer the instruments will be tested through interrater reliability.

**Intervention Group**

Participants in the intervention group will receive the mPEI programme for six weeks alongside the standard care they receive in the hospital.

**Control Group**

Participants in the control group will receive only the standard care in each hospital which entails routine care by doctors and nurses during their clinic attendance. Standard care is the control condition in the study. For each hospital, standard care will entail routine care by doctors and nurses during their clinic attendance. The standard care involves regular instructions on chemotherapy, side effects and general health education. The education takes place around 15 minutes during each clinic attendance.

***Outcome measures***

Data will be collected from the intervention and control group at baseline, and immediately after the completion of the intervention. All the instruments will be administered in English language which is the medium of instruction for the intervention.

*Self-efficacy for coping with cancer*

Self-efficacy for coping with cancer will be measured with the Cancer Behavior Inventory-Brief Version (CBI-B). The instrument comprises 12-items measuring confidence of individuals in performing activities that show how well they are coping with the demands of cancer and their treatment. Each item will be scored on a nine-point Likert scale with ‘1’ meaning ‘not at all confident’ and ‘9’ meaning ‘totally confident’. Higher scores indicate greater self-efficacy in coping. This instrument has been used in BC studies. It has also been validated and found reliable with a Cronbach’s α coefficient ranging from .84 to .88. (Heitzmann, 2001). (See Appendix C).

*Coping*

Coping will be assessed with Coping Orientation to Problems Experienced Inventory (Brief COPE). The scale can measure primary coping styles under three subscales: problem-focused coping, emotion-focused coping and avoidant coping. This 28-item instrument was developed as a short version of the original 60-item COPE scale (Carver et al., 1997). The 28 items are measured on a four-point scale (ranging from1= I haven’t been doing this at all, 2= a little bit, 3= a medium amount, and 4= I’ve been doing this a lot). The instrument has also been verified among patients with breast cancer receiving chemotherapy and have a fairly good reliability and validity. Internal consistencies ranged from 0.25 to 1.00, and intraclass correlation coefficient ranged between 0.05 and 1.00 (Yusoff et al., 2010). (See Appendix D).

*Anxiety and Depression*

Anxiety and depression will be assessed with the Hospital Anxiety and Depression Scale (HADS). The instrument has been used to measure anxiety and depression in BC patients. HADS comprises anxiety and depression subscales with seven items per subscale. A total score of 0-7 is normal, 8-10 is borderline abnormal and 11-21 is abnormal. This instrument has been validated and also found to be reliable with a Cronbach’s α coefficient of 0.79 for the anxiety subscale, 0.87 for the depression subscale, and 0.85 for all the items in an investigation into the psychometric properties in a previous study among BC patients (Rodgers et al., 2005) (See Appendix E).

*Symptom distress*

Symptom distress will be measured with the M. D. Anderson Symptom Inventory (MDASI) which comprises 13 items to measure symptom severity and six items to measure the rate of interference of the symptoms with patient’s activities of daily living. The symptom and interference items have been used in BC studies have been validated and found reliable with a Cronbach’s α coefficient of 0.85 and 0.91, respectively. (Cleeland, 2000). (See Appendix F).

*Quality of life*

Quality of life will be assessed using the Functional Assessment of Cancer Treatment-B (FACT-B). This instrument contains 37-items for measuring five domains of health-related quality of life in BC patients on a 5-point Likert scale with an alpha coefficient (internal consistency) of .90 (Brady et al., 1997). The total score for the five domains ranges from 0-148 with higher scores indicating better quality of life (See Appendix G).

***Demographic data sheet***

Demographic data will be collected on age, marital status, ethnic group, religion, level of education and occupation with a self-designed demographic data sheet (See Appendix H).

***Data collection***

The data collection will be conducted by two research assistants. Quantitative data will be conducted at baseline and immediately after the completion of the programme while qualitative data will be collected immediately after the programme completion. Data collection will be done at the oncology clinic. An information sheet which contains details of the intervention will be administered (Appendix I) after which the consent form will be completed (Appendix J).

The recruitment capability will be measured by documenting the recruitment rate, consent rate, and refusal rate based on the number of patients who are screened, those who are eligible, those who finally agree to be part of the study, and those who sign the informed consent form. Their reason for ineligibility and refusal to be part of the study will also be documented.

The acceptability and feasibility of the intervention will be assessed by documenting the attrition rate and the reason for lost to contact, adherence rate based on the number of participants that fully adhere to the protocol and data collection procedure, reasons for not adhering and any other adverse occurrence will be noted.

The acceptability of the intervention wi also be measured by a qualitative data collection. Three sessions of online, synchronous (live) focus group discussions with five participants in each group will be conducted by the research assistants. Online focus group discussion was considered appropriate for this part of the study due to its cost-effectiveness, no time commitment required for the participants to travel to the facility, ease of moderation and compatibility with the overall study design which is an online-delivered intervention (Rezabek, 2000). Questions for focus group interview in this phase is found in Appendix K. The focus group interview will explore the participants’ comments on acceptability of the intervention, ease of usage of the intervention and perceived usefulness of the intervention. Their findings will be used to improve the main intervention.

***Data analysis***

The quantitative data will be analysed using the SPSS version 25. The demographics and data related to intervention acceptability and feasibility will be analysed through descriptive statistics including frequencies, percentages, mean and standard deviation. Qualitative data will be analysed using NVIVO version 12. Thematic analysis will be conducted.

**MAIN RCT**

**Study design**

This study will be a multicentre RCT. This design was chosen for this study because RCTs are considered gold standard of experimental designs (Fewtrell et al., 2008) as they allow for random allocation which ensures equalisation of possible causes for intervention effects while allowing all subjects to have an equal chance of being assigned to a particular group (Polit & Beck, 2012). Also, RCTs help to establish the presumed cause-and-effect relationship between the study intervention and the outcomes which will help to generate knowledge that will be useful in clinical practice (Glasgow et al., 2003).

**Study setting**

Participants will be recruited from the oncology centers of Lagos State University Teaching Hospital (LASUTH), Ikeja and Lagos University Teaching Hospital (LUTH), Idi-Araba, Nigeria. At each hospital, approximately 50 women undergoing BC chemotherapy attend each center per month and an average of 25 of them have access to a smartphone and internet access.

**Sample**

Inclusion and exclusion criteria will be the same as the pilot study described in the previous phase.

***Sample size planning***

Sample size is calculated based on the primary outcome of self-efficacy. Power analysis was done with G*Power version 3.1 (Faul et al., 2007), and a medium effect size of 0.53 which was demonstrated in a previous mHealth psychoeducational study featuring women undergoing BC chemotherapy. Power was 0.8 and *p*<.05 with 10% attrition which was an average attrition rate found from a recent mhealth systematic review conducted on this population (Cohen 1988; Zhu et al., 2018). Sample size is estimated to be 126 participants, but the exact number will be confirmed after the pilot study.

**Outcomes measures**

The outcome measures will be the same as described in the pilot study.

**Fidelity of the intervention**

Fidelity of intervention refers to the degree of adherence to intervention protocol (Stein et al., 2007). To ensure fidelity in this study, the PI will obtain a certificate after participating in a training for health workers on psychoeducation for BC patients. The PI will also deliver all the sessions of the intervention. monitor and reinforce adherence to the program alongside provision of psychological support. This will ascertain consistency in the mode of delivery. The co-researchers that are experienced nurses working in the oncology units of the hospitals will provide nurse-consultancy services. The attendance rate of and response rate to the intervention will be monitored by the RAs. A personal link will be shared individually with the eligible participants to access the application. This link will be revoked after they are added. To prevent contamination, they will be instructed not to share the intervention content with any other patient. Process evaluation will be done to assess the fidelity of the intervention.

**Data collection procedure**

The data collection procedure will be the same as outlined in the pilot study. Flow chart for data collection is illustrated in the Consolidated Standards of Reporting Trials (CONSORT) in Appendix L.

***Participant recruitment***

The co-researchers who are nurses in the oncology units will introduce the mPEI programme to eligible women. For those interested, their eligibility will be confirmed by the co-researchers. They will be given detailed information about the study after which their consent will be sought to join the study. For those that consent, they will be required to complete and sign a consent form.

***Blinding***

The RAs will be blinded alongside the healthcare team. The patients will be aware of the intervention they are receiving, likewise the intervener (the researcher) will also be aware of the intervention; hence, they will not be blinded.

***Randomisation***

To reduce the chance for confounding, selection and accidental bias, while ascertaining that allocation of participants to the intervention and control groups is balanced (Efird, 2011); a block randomisation with a block size of four, six and eight using an allocation ratio of 1:1 will be conducted by an independent researcher who is not part of the research team. The participants will be randomised into two arms, intervention group who will receive the intervention and the control who will receive the standard care. Sealed sequentially numbered opaque envelopes will be assigned to each participant by the researcher.

**Process Evaluation**

As process evaluation is important in understanding and interpreting outcomes in trials (Khan et al., 2020), process evaluation will be conducted in this study to precisely determine how the intervention works and if this be the case, to further investigate how this intervention can be successfully implemented in standard clinical practice. The process evaluation will be guided by the Medical Research Council guidance for process evaluation of complex interventions (Moore et al., 2015). The three components emphasised in the framework as essential to understanding intervention outcomes will be explored which include: implementation, mechanisms of impact and context (Moore et al., 2015).

The objectives for the evaluation are to: (i) assess the dose and reach of the intervention, ii) assess the adaptability of the intervention features for individual needs of the participants, (iii) explore the mPEI program from the perspective of the participants and the nurses to gain a deeper insight into mechanisms underlying changes in outcomes while probing unexpected consequences, (iv) evaluate external factors that might have affected intervention delivery, and (v) investigate resources and implementation processes required for effective clinical implementation of the intervention. The convergence model by Creswell and Plano Clark, (2011) will be utilised during the process evaluation for analysis of quantitative and qualitative data.

**Data analysis**

SPSS version 25 will be used to analyse quantitative data. The demographic data and clinical data will be analysed through descriptive statistics including frequencies, percentages, mean and standard deviation. Continuous variables at baseline, and immediately after the intervention will be compared through independent samples t- test for the continuous variables and chi-square or Fisher’s exact test for the categorical variables where appropriate. Primary and secondary outcomes data will be analysed through the generalized estimating equation (GEE) model provided the assumptions for homogeneity of variance and normality are met (Bell et al., 2014). In a bid to control for attrition bias, the intention-to-treat principle will be used to account for loss to follow up (Nam & Toneatto, 2016).

**Ethical considerations**

The ethical principles of the Declaration of Helsinki (World Medical Association, 2013) will be adhered to in this study. Ethical approval will be sought from the Joint Chinese University of Hong Kong-New Territories East Cluster Clinical Research Ethics Committee and the study settings in Nigeria. Approval to use the instruments will be obtained from the original authors. All through the phases, the research assistants will administer an information sheet containing detailed information about the study and they will complete a consent form afterwards.

The participants will be assured that the data collected will be used solely for the research and they will also be given a chance to ask questions and make clarifications. They will also be informed that they do not need to pay to use the application. Similarly, they will be assured that participation is voluntary and their decision to participate or otherwise will not affect their treatment. Similarly, they will be assured of confidentiality through the study.

During interaction on the mobile app, the participants will be free to use a name they are comfortable with or remain anonymous so their identity can be confidential. They will also be informed not to share the details of their interaction with anybody who is not part of the intervention to maintain privacy. Furthermore, they will not be required to display their personal pictures. Sensitive issues will not be raised during the interaction on the application. The application will not made available on social networking sites or public domains. Each participant will be given a unique link to download the application. After download, the link will be revoked. Each participant will create a unique username and password which they will be instructed not to share with any other person. They will be able to change the password anytime they desire. Data from the app will be encrypted and will only be accessible to the principal investigator and software engineer. The participants will be informed that the principal investigator and software engineer can access their profile. The principal investigator and the software engineer will be responsible for safekeeping of the data from the application during the study. Other study data will be safely kept by the principal investigator. The software engineer will transfer the data from the application to the principal investigator and supervisor after the study. The principal investigator will be responsible for keeping all study data in a locked cabinet after the study. All paper copies of the data will be destroyed by a paper shredder and all electronic copies will be permanently deleted from the computer and storage devices by the principal investigator five years after the study is completed and published. Principles of beneficence, non-maleficence, and trust will be upheld throughout the study.

**Impact of the study on Nursing Practice**

As the usage of mHealth in this population is relatively new and with very limited evidence; this study, which to the best of the researcher’s knowledge is the first in Nigeria, will provide evidence for the integration of mHealth into BC chemotherapy. Similarly, nurses will be informed about the effectiveness of mPEI for this population. Finally, it is hoped that the psychological concerns of Nigerian women with BC will be adequately addressed while receiving chemotherapy.

**References**

Anarado, A. N., Ezeome, E. R., Ofi, O. B., Nwaneri, A. C., & Ogbolu, Y. (2017). Experiences and desired nursing assistance of women on out-patient breast cancer chemotherapy in Southeastern Nigeria. *Psycho-Oncology*, *26*(3), 385–391. doi.org/10.1002/pon.4034

Akin, S., Can, G., Durna, Z., & Aydiner, A. (2008). The quality of life and self-efficacy of Turkish breast cancer patients undergoing chemotherapy. *European Journal of Oncology Nursing, 12*(5), 449–456. doi:10.1016/j.ejon.2008.07.006

Akin-Odanye, E. O., Asuzu Chioma, C., & Popoola Abiodun, O. (2011). Measured effect of some socio-demographic factors on depression among breast cancer patients receiving chemotherapy in Lagos State University Teaching Hospital (LASUTH). *African Health Sciences*, *11*(3), 341-345. <https://www.ncbi.nlm.nih.gov/pmc/articles/PMC3261007/>

Akhtar, M. (2008)*.* What is self-efficacy? Bandura’s 4 sources of efficacy beliefs. Positive Psychology UK. http://positivepsychology.org.uk/self-efficacy-definition-bandura meaning

Aranda-jan Clara, Mohutsiwa-dibe Neo, & Svetla, L. (2014). Systematic review on implementation of mobile health projects in Africa: What works? What doesn’t work and why? *BMC Public Health*, *14*, 2-15. doi: 10.1186/1471-2458-14-188

Arcas, M.M., Buron, A., Ramis, O., Esturi, M., Hernández, C., Macià, F. (2014). Can a mobile phone short message increase participation in breast cancer screening programmes? *Revista de Calidad Asistencial*, *29*(4), 188–96. doi: 10.1016/j.cali.2014.02.003

Ashing, K. T., & George, M. (2020). Exploring the efficacy of a paraprofessional delivered telephonic psychoeducational intervention on emotional well-being in African American breast cancer survivors. *Supportive Care in Cancer*, *28*(3), 1163–1171. https://doi.org/10.1007/s00520-019-04899-7

Au, A., Lam, W., Tsang, J., Yau, T., Soong, I., Yeo, W., … Fielding, R. (2012). Supportive care needs in Hong Kong Chinese women confronting advanced breast cancer. *Psycho-Oncology,* 22(5), 1144–1151. doi:10.1002/pon.3119

Azubuike, S. O., Muirhead, C., Hayes, L., & McNally, R. (2018). Rising global burden of breast cancer: the case of sub-Saharan Africa (with emphasis on Nigeria) and implications for regional development: a review. *World Journal of Surgical Oncology, 16*(1), 63. https://doi.org/10.1186/s12957-018-1345-2

Bandura, A (1977). Self-efficacy: Toward a Unifying Theory of Behavioral Change. *Psychological Review 84* (2): 191–215

Bandura, A. (1989). Human agency in social cognitive theory. *American Psychologist, 44,* 1175–1184.

Bandura, A. (1995)*. Self-efficacy in changing societies*. New York: Cambridge University.

Bandura, A. (1997). *Self-efficacy: The exercise of control.* New York: Freeman.

Bandura, A*.* (2004).  *Health promotion by social cognitive means. Health Education and Behavioir 31*(2), 143-64.

Barsevick, A. M., Sweeney, C., Haney, E., & Chung, E. (2002). A Systematic Qualitative Analysis of Psychoeducational Interventions for Depression in Patients With Cancer. *Oncology Nursing Forum, 29*(1), 73–87.

Bäuml, J., Froböse, T., Kraemer, S., Rentrop, M., & Pitschel-Walz, G. (2006). Psychoeducation: a basic psychotherapeutic intervention for patients with schizophrenia and their families. *Schizophrenia bulletin, 32 Suppl 1*(Suppl 1), S1–S9. https://doi.org/10.1093/schbul/sbl017

Beauchamp, A., Mohebbi, M., Cooper, A., Pridmore, V., Livingston, P., Scanlon, M., Davis, M., O’Hara, J., & Osborne, R. (2020). The impact of translated reminder letters and phone calls on mammography screening booking rates: Two randomised controlled trials. *PLoS ONE*, *15*(1), 1–16. https://doi.org/10.1371/journal.pone.0226610

Bell, M. L., Fiero, M., Horton, N. J., & Hsu, C. H. (2014). Handling missing data in RCTs: A review of the top medical journals. *BMC Medical Research Methodology*, *14*(1), 1–8. https://doi.org/10.1186/1471-2288-14-118

Bender, J.L., Yue, R.Y., To, M.J., Deacken. L. Jadad ,A.R. (2013). A Lot of Action, But Not in the Right Direction: Systematic Review and Content Analysis of Smartphone Applications for the Prevention, Detection, and Management of Cancer. *Journal of Medical Internet Research, 15*(12), e287. doi: https://doi.org/10.2196/jmir.2661

Biggs, K., Hind, D., Gossage-Worrall, R., Sprange, K., White, D., Wright, J., Chatters, R., Berry, K., Papaioannou, D., Bradburn, M., Walters, S. J., & Cooper, C. (2020). Challenges in the design, planning and implementation of trials evaluating group interventions. *Trials, 21*(1), 116. https://doi.org/10.1186/s13063-019-3807-4

Bloor, M., Frankland, J., Thomas, M., & Robson, K. (2001). *Focus groups in social research*. Thousand Oaks, CA: Sage Publications Inc.

BorjAlilu, S., Kaviani, A., Helmi, S., Karbakhsh, M., & Mazaheri, M. A. (2017). Exploring the Role of Self-Efficacy for Coping With Breast Cancer: A Systematic Review. *Archives of Breast Cancer, 4*(2), 42–57. https://doi.org/10.19187/abc.20174242-57

Børøsund, E., Ehlers, S. L., Varsi, C., Clark, M. M., Andrykowski, M. A., Cvancarova, M., & Solberg Nes, L. (2020). Results from a randomized controlled trial testing StressProffen; an application-based stress-management intervention for cancer survivors. *Cancer Medicine*, *9*(11), 3775–3785. https://doi.org/10.1002/cam4.3000

Brady, M. J., Cella, D. F., Mo, F., Bonomi, A. E., Tulsky, D. S., Lloyd, S. R., Deasy, S., Cobleigh, M., & Shiomoto, G. (1997). Reliability and validity of the Functional Assessment of Cancer Therapy-Breast quality-of-life instrument. *Journal of Clinical Oncology 15*(3), 974–986. https://doi.org/10.1200/JCO.1997.15.3.974

Browall, M., Brandberg, Y., Nasic, S., Rydberg, P., Bergh, J., Rydén, A., Xie, H., Eriksson, I., & Wengström, Y. (2017). A prospective exploration of symptom burden clusters in women with breast cancer during chemotherapy treatment. *Supportive Care in Cancer, 25*(5), 1423–1429. https://doi.org/10.1007/s00520-016-3527-1

Burgess, C., Cornelius, V., Love, S., Graham, J., Richards, M., & Ramirez, A. (2005). Depression and anxiety in women with early breast cancer: five year observational cohort study. *BMJ (Clinical research ed.), 330* (7493), 702. https://doi.org/10.1136/bmj.38343.670868.D3

Callahan,C.M., Unverzagt, F.W., Hui, S.L.,Perkins, A.J., Hendrie, H.C. (2002). Six-Item Screener to Identify Cognitive Impairment among Potential Subjects for Clinical Research. *Medical Care* *(40) 9*, 771-781. https://www.jstor.org/stable/3768143

Carlson, R. W., Allred, D. C., Anderson, B. O., Burstein, H. J., Carter, W. B., Edge, S. B., … Wolff, A. C. (2009). *Breast Cancer. Journal of the National Comprehensive Cancer Network, 7*(2), 122–192. doi:10.6004/jnccn.2009.0012

Charfare, H., Limongelli, S., & Purushotham, A. D. (2005). Neoadjuvant chemotherapy in breast cancer. *British Journal of Surgery, 92*(1), 14–23. doi:10.1002/bjs.4840

Chen, Y. Y., Guan, B. S., Li, Z. K., & Li, X. Y. (2018). Effect of telehealth intervention on breast cancer patients’ quality of life and psychological outcomes: A meta-analysis. *Journal of Telemedicine and Telecare*, *24*(3), 157- 167. https://doi.org/10.1177/1357633X16686777

Chesney, M. A., Neilands, T. B., Chambers, D. B., Taylor, J. M., & Folkman, S. (2006). A validity and reliability study of the coping self-efficacy scale. *British Journal of Health Psychology, 11*(3), 421–437. https://doi.org/10.1348/135910705X53155

Chow, K. M., Chan, J. C. Y., Choi, K. K. C., & Chan, C. W. H. (2016). A review of psychoeducational interventions to improve sexual functioning, quality of life, and psychological outcomes in gynecological cancer patients. *Cancer Nursing*, *39*(1), 20–31. https://doi.org/10.1097/NCC.0000000000000234

Chu, E. (Ed.). (2018). Cancer Chemotherapy In Katzung, B.G. Basic & Clinical Pharmacology (14^th^ ed.). Lange.

Cleeland, C. S., Mendoza, T. R., Wang, X. S., Chou, C., Harle, M. T., Morrissey, M., & Engstrom, M. C. (2000). Assessing symptom distress in cancer patients: the M.D. Anderson Symptom Inventory. *Cancer, 89*(7), 1634–1646. https://doi.org/10.1002/1097-0142(20001001)89:7<1634::aid-cncr29>3.0.co;2-v

Cohen, F. (1984). Coping. In J.D. Matarazzo, N.E., C.M. Weiss, J.A. Herd, N.E. Miller, & S.M. Weiss (Eds.), Behavioral health: A hand- book of health enhancement and disease prevention (pp. 261–270). New York, NY: John Wiley and Sons.

Cohen J. (1988). The analysis of variance and covariance. In: N.J. Hillsdale *Statistical Power Analysis for the Behavioral Sciences* (2^nd^ ed.). (pp. 273-406). Lawrence Erlbaum Associates.

Collins, W.L., & Antle, B.F. (2010). African American women living beyond breast cancer in a Kentucky support group. Social Work and Christianity, 37, 65–77

Creswell, J. W. (2007). *Designing and conducting mixed methods research*. Thousand Oaks, Calif: SAGE Publications.

David, E., (2003). Hierarchy of evidence: a framework for ranking evidence evaluating healthcare interventions. *Journal of Clinical Nursing* *12*(1),77–84. doi:10.1046/j.1365-2702. 2003.00662.

Davis, S. W., & Oakley-Girvan, I. (2015). mHealth Education Applications Along the Cancer Continuum. *Journal of Cancer Education, 30*(2), 388–394. https://doi.org/10.1007/s13187-014-0761-4

Deeks, J.J., Higgins, J.P., Altman, D.G. (Eds.). (2021). Analysing data and undertaking meta-analyses. In J.P. Higgins, J. Chandler, M. Cumpston, T. Li, M.J. Page, V.A. Welch*.* Cochrane Handbook for Systematic Reviews of Interventions version 6.2. Cochrane.

Diaby, V., Tawk, R., Sanogo, V., Xiao, H., & Montero, A. J. (2015). A review of systematic reviews of the cost-effectiveness of hormone therapy, chemotherapy, and targeted therapy for breast cancer. *Breast Cancer Research and Treatment, 151*(1), 27–40. doi:10.1007/s10549-015-3383-6

Denieffe, S., & Gooney, M. (2011). A meta-synthesis of women’s symptoms experience and breast cancer. E*uropean Journal of Cancer Care, 20*(4), 424–435. https://doi.org/10.1111/j.1365-2354.2010.01223.x

Devonport, T., & Lane, A. (2004). Exploring the relationship between self-efficacy and coping amongst undergraduate students. *Learning and Teaching Projects- University of Wolverhampton* https://core.ac.uk/download/pdf/1931624.pdf

Diaby, V., Tawk, R., Sanogo, V., Xiao, H., & Montero, A. J. (2015). A review of systematic reviews of the cost-effectiveness of hormone therapy, chemotherapy, and targeted therapy for breast cancer. *Breast cancer research and treatment, 151*(1), 27–40. https://doi.org/10.1007/s10549-015-3383-6

Dorri, S., Asadi, F., Olfatbakhsh, A., & Kazemi, A. (2020). A Systematic Review of Electronic Health (eHealth) interventions to improve physical activity in patients with breast cancer. *Breast Cancer*, *27*(1), 25–46. https://doi.org/10.1007/s12282-019-00982-3

Du, X., Goodwin, J.S. (2001). Patterns of Use of Chemotherapy for Breast Cancer in Older Women: Findings From Medicare Claims Data. *Journal of Clinical Oncology, 19*(5) http://citeseerx.ist.psu.edu/viewdoc/download?doi=10.1.1.978.4835&rep=rep1&type=pdf

Efird, J. (2011). Blocked randomization with randomly selected block sizes. *International Journal of Environmental Research and Public Health*, *8*(1), 15–20. https://doi.org/10.3390/ijerph8010015

Ekhtiari, H., Rezapour, T., Aupperle, R. L., & Paulus, M. P. (2017). Neuroscience-informed psychoeducation for addiction medicine: A neurocognitive perspective. Progress in brain research, 235, 239–264. https://doi.org/10.1016/bs.pbr.2017.08.013

Eng, D. S., & Lee, J. M. (2013). The promise and peril of mobile health applications for diabetes and endocrinology. *Pediatric diabetes, 14*(4), 231–238. https://doi.org/10.1111/pedi.12034

Fan, L., Strasser-Weippl, K., Li, J. J., St Louis, J., Finkelstein, D. M., Yu, K. Da, Chen, W. Q., Shao, Z. M., & Goss, P. E. (2014). Breast cancer in China. *The Lancet Oncology, 15*(7). https://doi.org/10.1016/S1470-2045(13)70567-9

Fanakidou, I., Zyga, S., Alikari, V., Tsironi, M., Stathoulis, J., & Theofilou, P. (2017). Mental health, loneliness, and illness perception outcomes in quality of life among young breast cancer patients after mastectomy: the role of breast reconstruction. *Quality of Life Research, 27*(2), 539–543. doi:10.1007/s11136-017-1735-x

Fann, J. R., Thomas-Rich, A. M., Katon, W. J., Cowley, D., Pepping, M., McGregor, B. A., & Gralow, J. (2008). Major depression after breast cancer: a review of epidemiology and treatment. *General hospital psychiatry, 30*(2), 112–126. https://doi.org/10.1016/j.genhosppsych.2007.10.008

Fatiregun, O. A., Olagunju, A. T., Erinfolami, A. R., Fatiregun, O. A., Arogunmati, O. A., & Adeyemi, J. D. (2016). Anxiety disorders in breast cancer: Prevalence, types, and determinants. Journal of Psychosocial Oncology, 34(5), 432–447. https://doi.org/10.1080/07347332.2016.1196805

Faul, F., Erdfelder, E., Lang, A.-G., & Buchner, A. (2007). G*Power 3: A flexible statistical power analysis program for the social, behavioral, and biomedical sciences. *Behavior Research Methods, 39*, 175-191.

Federal Ministry of Health (2018). National Cancer Control Plan- Nigeria 2018-2022. https://www.iccp-portal.org/system/files/plans/NCCP_Final%20%5B1%5D.pdf

Fewtrell, M. S., Kennedy, K., Singhal, A., Martin, R. M., Ness, A., Hadders-Algra, M., Koletzko, B., & Lucas, A. (2008). How much loss to follow-up is acceptable in long-term randomised trials and prospective studies? *Archives of Disease in Childhood*, *93*(6), 458–461. https://doi.org/10.1136/adc.2007.127316

Fjell, M., Langius-Eklöf, A., Nilsson, M., Wengström, Y., & Sundberg, K. (2020). Reduced symptom burden with the support of an interactive app during neoadjuvant chemotherapy for breast cancer – A randomized controlled trial. *Breast*, *51*, 85–93. https://doi.org/10.1016/j.breast.2020.03.004

Gaston-Johansson, F., Haisfield-Wolfe, M. E., Reddick, B., Goldstein, N., & Lawal, T. A. (2013). The Relationships Among Coping Strategies, Religious Coping, and Spirituality in African American Women With Breast Cancer Receiving Chemotherapy. *Oncology Nursing Forum, 40*(2)*,* 120–131. doi:10.1188/13.onf.120-131

Gaston-Johansson, F., Fall-Dickson, J.M., Nanda, J.P., Sarenmalm, E.K., Browall, M., & Goldstein, N. (2013). Long-term effect of the self-management Comprehensive Coping Strategy Program on quality of life in patients with breast cancer treated with high-dose chemotherapy. Psycho-Oncology, 22, 530–539. https://doi .org/10.1002/pon.3031

Galiano-Castillo, N., Arroyo-Morales, M., Lozano-Lozano, M., Fernández-Lao, C., Martín-Martín, L., Del-Moral-Ávila, R., & Cantarero-Villanueva, I. (2017). Effect of an Internet-based telehealth system on functional capacity and cognition in breast cancer survivors: a secondary analysis of a randomized controlled trial. *Supportive Care in Cancer*, *25*(11), 3551–3559. https://doi.org/10.1007/s00520-017-3782-9

Gallia, K.S., & Pines, E.W. (2009). Narrative identity and spirituality of African American churchwomen surviving breast cancer survivors. *Journal of Cultural Diversity, 16,* 50–55.

Ghanbari, E., Yektatalab, S., & Mehrabi, M. (2021). Effects of Psychoeducational Interventions Using Mobile Apps and Mobile-Based Online Group Discussions on Anxiety and Self-Esteem in Women With Breast Cancer: Randomized Controlled Trial. *JMIR mHealth and uHealth, 9*(5), e19262. https://doi.org/10.2196/19262

Giunti, G., Giunta, D. H., Guisado-Fernandez, E., Bender, J. L., & Fernandez-Luque, L. (2018). A biopsy of Breast Cancer mobile applications: state of the practice review. *International Journal of Medical Informatics*, *110*(December), 1–9. https://doi.org/10.1016/j.ijmedinf.2017.10.022

Glanz, K., Rimer, B. k., & Viswanath, K. (2002). *Health Behaviour and Health Education- Theory Research and Practice*. John Wiley & Sons, Inc. USA

Glasgow, R. E., Lichtenstein, E., & Marcus, A. C. (2003). Why don’t we see more translation of health promotion research to practice? rethinking the efficacy-to-effectiveness transition. *Journal Information, 93*(8), 1261-1267.

Global Cancer Observatory (2020) New Global Cancer Data https://www.uicc.org/news/globocan-2020-new-global-cancer-data

Gold, L. S., Lee, C. I., Devine, B., Nelson, H., Chou, R., Ramsey, S., & Sullivan, S. D. (2014). Imaging Techniques for Treatment Evaluation for Metastatic Breast Cancer. 1. https://pubmed.ncbi.nlm.nih.gov/25375016%0Ahttps://www.ncbi.nlm.nih.gov/books/NBK253155

Greenberg, D. (2010). Psychological Support for the Breast Cancer Patient. In: I. Jatoi, M. Kaufmann (eds.), Management of Breast Diseases. Springer, Berlin, Heidelberg. https://doi.org/10.1007/978-3-540-69743-5_28

Guarino, A., Polini, C., Forte, G., Favieri, F., Boncompagni, I., & Casagrande, M. (2020). The Effectiveness of Psychological Treatments in Women with Breast Cancer: A Systematic Review and Meta-Analysis. *Journal of Clinical Medicine*, *9*(1), 209. https://doi.org/10.3390/jcm9010209

Guo, Z., Tang, H. ying, Li, H., Tan, S. kui, Feng, K. hua, Huang, Y. chun, Bu, Q., & Jiang, W. (2013). The benefits of psychosocial interventions for cancer patients undergoing radiotherapy. *Health and Quality of Life Outcomes*, *11*(1), 1–12. https://doi.org/10.1186/1477-7525-11-121

Gupta, A., Ocker, G., & Chow, P. I. (2020). Recruiting breast cancer patients for mHealth research: Obstacles to clinic-based recruitment for a mobile phone app intervention study. *Clinical Trials*. https://doi.org/10.1177/1740774520939247

Hamilton, J.B., Powe, B.D., Pollard, A.B., 3rd, Lee, K.J., & Felton, A.M. (2007). Spirituality among African American cancer survivors: Having a personal relationship with God. Cancer Nursing, 30, 309–316. doi:10.1097/01.NCC.0000281730.17985.f5

Handa, S., Okuyama, H., Yamamoto, H., Nakamura, S., & Kato, Y. (2020). Effectiveness of a Smartphone Application as a Support Tool for Patients Undergoing Breast Cancer Chemotherapy: A Randomized Controlled Trial. Clinical Breast Cancer, 20(3), 201–208. https://doi.org/10.1016/j.clbc.2020.01.004

Hajian, S., Mehrabi, E., Simbar, M., & Houshyari, M. (2017). Coping Strategies and Experiences in Women with a Primary Breast Cancer Diagnosis. *Asian Pacific Journal of Cancer Prevention* : APJCP, 18(1), 215–224. https://doi.org/10.22034/APJCP.2017.18.1.215

Heitzmann, C.A., Merluzzi, T. V., Nairn, R. C., Hegde, K., Martinez Sanchez, M. A., & Dunn, L. (2001). Self-efficacy for coping with cancer: revision of the Cancer Behavior Inventory (version 2.0). *Psycho-oncology, 10*(3), 206–217. https://doi.org/10.1002/pon.511

Hewitt, M., Herdman, R., Holland, J. (2004). *Meeting Psychosocial Needs of Women with Breast Cancer.* National Academic Press: Washington, DC.

Henderson, V.P., Clemow, L., Massion, A.O., Hurley, T.G., Druker, S., & Hébert, J.R. (2012). The effects of mindfulness-based stress reduction on psychosocial outcomes and quality of life in early stage breast cancer patients: A randomized trial. *Breast Cancer Research and Treatment, 131*, 99–109. https://doi.org/10.1007/s10549-011-1738-1

Hohenthal, J., Owidi, E., Minoia, P., & Pellikka, P. (2015). Local assessment of changes in water‐related ecosystem services and their management: DPASER conceptual model and its application in Taita Hills, Kenya*. International Journal of Biodiversity Science, Ecosystem Services & Management, 11*, 225– 238.

Hou, I. C., Lin, H. Y., Shen, S. H., Chang, K. J., Tai, H. C., Tsai, A. J., & Dykes, P. C. (2020). Quality of life of women after a first diagnosis of breast cancer using a self-management support mHealth app in Taiwan: Randomized controlled trial. *JMIR MHealth and UHealth*, *8*(3), 1–13. https://doi.org/10.2196/17084

Javadi, P., Nejat, N., Golaghaie, F., & Sharifi, M. (2019). The effects of nurse-led telephone-based support on supportive care needs among women with breast cancer: A randomized clinical trial. *Nursing and Midwifery Studies*, *8*(1), 7–13. doi: 10.4103/nms.nms_94_17

Joanna Briggs Institute (2021). Checklist for Randomized Controlled Trials. The University of Adelaide https://jbi.global/critical-appraisal-tools

Johnson, R. B., & Onwuegbuzie, A. J. (2004). Mixed methods research: a research paradigm whose time has come. *Educational Researcher, 33*(7), 14–26.

Jongerius, C., Russo, S., https://doi.org/10.4103/nms.nms_94_17Mazzocco, K., & Pravettoni, G. (2019). Research-Tested Mobile Apps for Breast Cancer Care: Systematic Review. *JMIR MHealth and UHealth*, *7*(2), e10930–e10930. https://doi.org/10.2196/10930

Kato, Y., Kageyama, K., Mesaki, T., Uchida, H., Sejima, Y., Marume, R., Takahashi, K., & Hirao, K. (2020). Study protocol for a pilot randomized controlled trial on a smartphone application-based intervention for subthreshold depression: Study protocol clinical trial (SPIRIT Compliant). *Medicine, 99*(4), e18934. https://doi.org/10.1097/MD.0000000000018934

Kerrison, R. S., Shukla, H., Cunningham, D., Oyebode, O., & Friedman, E. (2015). Text-message reminders increase uptake of routine breast screening appointments: A randomised controlled trial in a hard-to-reach population. *British Journal of Cancer*, *112*(6), 1005–1010. https://doi.org/10.1038/bjc.2015.36

Kim, H. J., Kim, S. M., Shin, H., Jang, J. S., Kim, Y. I., & Han, D. H. (2018). A mobile game for patients with breast cancer for chemotherapy self-management and quality-of-life improvement: Randomized controlled trial. *Journal of Medical Internet Research*, *20*(10), 1–10. https://doi.org/10.2196/jmir.9559

Krueger, R. A., & Casey, M. A. (2000). *Focus groups: A practical guide for applied research,* 4th ed. Thousand Oaks, CA: Sage Publications Inc.

Lazarus, R.S., Folkman, S (1984). *Stress, appraisal, and coping*. New York: Springer..

Loiselle, C. G., Edgar, L., Batist, G., Lu, J., & Lauzier, S. (2010). The impact of a multimedia informational intervention on psychosocial adjustment among individuals with newly diagnosed breast or prostate cancer: a feasibility study. *Patient education and counseling, 80*(1), 48–55. https://doi.org/10.1016/j.pec.2009.09.026

Lawlor, D. A., Tilling, K., & Davey Smith, G. (2017). Triangulation in aetiological epidemiology. *International Journal of Epidemiology*, *45*(6) https://doi.org/10.1093/ije/dyw314

Lee, H., Ghebre, R., Le, C., Jang, Y. J., Sharratt, M., & Yee, D. (2017). Mobile Phone Multilevel and Multimedia Messaging Intervention for Breast Cancer Screening: Pilot Randomized Controlled Trial. *JMIR MHealth and UHealth*, *5*(11), e154. https://doi.org/10.2196/mhealth.7091

Lee, M. K., Yun, Y. H., Park, H. A., Lee, E. S., Jung, K. H., & Noh, D. Y. (2014). A Web-based self-management exercise and diet intervention for breast cancer survivors: Pilot randomized controlled trial. *International Journal of Nursing Studies*, *51*(12), 1557–1567. https://doi.org/10.1016/j.ijnurstu.2014.04.012

Lepore, S. J., Buzaglo, J. S., Lieberman, M. A., Golant, M., Greener, J. R., & Davey, A. (2014). Comparing standard versus prosocial internet support groups for patients with breast cancer: A randomized controlled trial of the helper therapy principle. *Journal of Clinical Oncology*, *32*(36), 4081–4086. https://doi.org/10.1200/JCO.2014.57.0093

Lo, C., Liao, F. Z., & Chen, S. L. (2017). Symptom experiences of breast cancer patients. *Journal of Nursing, 64*(2), 19–27. https://doi.org/10.6224/JN.000021

Lukens,E.P. & McFarlane, W.R. (2004). Psychoeducation as evidence-based practice: considerations for practice, research, and policy. *Brief Treat Crisis Intery 4*(3), 205.

Manojlovich, M., & Sidani, S. (2008). Nurse dose: what's in a concept?. *Research in Nursing & Health, 31*(4), 310–319. https://doi.org/10.1002/nur.20265

Matsen, C. B., & Neumayer, L. A. (2013). Breast cancer: a review for the general surgeon. *JAMA surgery, 148*(10), 971–979. https://doi.org/10.1001/jamasurg.2013.3393

Maughan, K. L., Lutterbie, M. A., & Ham, P. S. (2010). Treatment of breast cancer. *American family physician, 81*(11), 1339–1346.

Medical Research Council (MRC), 2000*. A Framework for the Development and Evaluation of RCTs for Complex Interventions to Improve Health*. Medical Research Council, London, United Kingdom.

Mobasheri, M. H., Johnston, M., King, D., Leff, D., Thiruchelvam, P., & Darzi, A. (2014). Smartphone breast applications - What’s the evidence? *Breast*, *23*(5), 683–689. https://doi.org/10.1016/j.breast.2014.07.006

Moghaddasi, H., Asadi, F., Hosseini, A., & Ebnehoseini, Z. (2012). E-health: A global approach with extensive semantic variation. *Journal of Medical Systems*, *36*(5), 3173–3176. https://doi.org/10.1007/s10916-011-9805-z

Moore (2021). How to Design Psychoeducational Interventions With Quenza. https://positivepsychology.com/quenza-psychoeducation-interventions/

Moyer, A., Sohl, S. J., Knapp-Oliver, S. K., & Schneider, S. (2009). Characteristics and methodological quality of 25 years of research investigating psychosocial interventions for cancer patients. *Cancer Treatment Reviews*, *35*(5), 475–484. https://doi.org/10.1016/j.ctrv.2009.02.003

Nam, S., & Toneatto, T. (2016). The Influence of Attrition in Evaluating the Efficacy and Effectiveness of Mindfulness-Based Interventions. *International Journal of Mental Health and Addiction*, *14*(6), 969–981. https://doi.org/10.1007/s11469-016-9667-1

National Breast Cancer Centre (2003). Clinical Practice Guidelines for the Psychosocial Care of Adults with Cancer. https://www.canceraustralia.gov.au/sites/default/files/publications/pca-1-clinical-practice-guidelines-for-psychosocial-care-of-adults-with-cancer_504af02682bdf.pdf

Neugut, A. I., Hillyer, G. C., Kushi, L. H., Lamerato, L., Buono, D. L., Nathanson, S. D., Bovbjerg, D. H., Mandelblatt, J. S., Tsai, W. Y., Jacobson, J. S., & Hershman, D. L. (2016). A prospective cohort study of early discontinuation of adjuvant chemotherapy in women with breast cancer: the breast cancer quality of care study (BQUAL). *Breast Cancer Research and Treatment*, *158*(1), 127–138. https://doi.org/10.1007/s10549-016-3855-3

Ohaeri, B. M., Ofi, A. B., & Campbell, O. B. (2012). Relationship of knowledge of psychosocial issues about cancer with psychic distress and adjustment among breast cancer clinic attendees in a Nigerian teaching hospital. Psycho-Oncology, 21(4), 419–426. https://doi.org/10.1002/pon.1914

Okunade, K. S., Salako, O., Adejimi, A. A., Akinsola, O. J., Fatiregun, O., Adenekan, M. A., Moses, O. E., Ebenso, B., Allsop, M. J., Anorlu, R. I., & Berek, J. S. (2020). Impact of mobile technologies on cervical cancer screening practices in Lagos, Nigeria (mHealth-Cervix): Protocol for a randomised controlled trial. *F1000Research*, *9*, 1–9. https://doi.org/10.12688/f1000research.22991.1

Oncology Nursing Society (2018). Psychoeducation/Psychoeducational interventions. https://www.ons.org/node/901?display=pepnavigator&sort_by=created&items_per_page=5

Onwuegbuzie, A. J., Dickinson, W. B., Leech, N. L., & Zoran, A. G. (2009). A Qualitative Framework for Collecting and Analyzing Data in Focus Group Research. *International Journal of Qualitative Methods, 8*(3), 1–21. doi:10.1177/160940690900800301

Reed, D., Titler, M. G., Dochterman, J. M., Shever, L. L., Kanak, M., & Picone, D. M. (2007). Measuring the dose of nursing intervention. *International Journal of Nursing Terminologies and Classifications 18*(4), 121–130. https://doi.org/10.1111/j.1744-618X.2007.00067.x

Reich, M., Lesur, A., & Perdrizet-Chevallier, C. (2008). Depression, quality of life and breast cancer: a review of the literature. *Breast cancer research and treatment, 110*(1), 9–17. https://doi.org/10.1007/s10549-007-9706-5

Rodgers, J., Martin, C. R., Morse, R. C., Kendell, K., & Verrill, M. (2005). An investigation into the psychometric properties of the Hospital Anxiety and Depression Scale in patients with breast cancer. *Health and quality of life outcomes*, 3, 41. https://doi.org/10.1186/1477-7525-3-41

Rosser, B. A., & Eccleston, C. (2011). Smartphone applications for pain management. *Journal of telemedicine and telecare, 17*(6), 308–312. https://doi.org/10.1258/jtt.2011.101102

Rottmann, N., Dalton, S. O., Christensen, J., Frederiksen, K., & Johansen, C. (2010). Self-efficacy, adjustment style and well-being in breast cancer patients: a longitudinal study. *Quality of life research*, *19*(6), 827–836. https://doi.org/10.1007/s11136-010-9653-1

Salehi, F., Turner, M. C., Phillips, K. P., Wigle, D. T., Krewski, D., & Aronson, K. J. (2008). Review of the Etiology of Breast Cancer with Special Attention to Organochlorines as Potential Endocrine Disruptors. *Journal of Toxicology and Environmental Health*, *11*(3-4), 276–300. doi:10.1080/10937400701875923

Sharma, G. N., Dave, R., Sanadya, J., Sharma, P., & Sharma, K. K. (2010). Various types and management of breast cancer: an overview. J*ournal of Advanced Pharmaceutical Technology & Research, 1*(2), 109–126. https://www.ncbi.nlm.nih.gov/pmc/articles/PMC3255438/

Silva, S.M., Crespo, C., & Canavarro, M.C. (2012). Pathways for psychosocial adjustment in breast cancer: A longitudinal study on coping strategies and posttraumatic growth. *Psychology and Health, 27,* 1323–1341. https://doi.org/10.1080/08870446.2012.676644

Sin, J., & Norman, I. (2013). Psychoeducational interventions for family members of people with schizophrenia: a mixed-method systematic review. *The Journal of Clinical Psychiatry, 74*(12), e1145–e1162. https://doi.org/10.4088/JCP.12r08308

Sin, J., Gillard, S., Spain, D., Cornelius, V., Chen, T., & Henderson, C. (2017). Effectiveness of psychoeducational interventions for family carers of people with psychosis: A systematic review and meta-analysis. *Clinical Psychology Review, 56*, 13–24. https://doi.org/10.1016/j.cpr.2017.05.002

So, W. K. W., Marsh, G., Ling, W. M., Leung, F. Y., Lo, J. C. K., Yeung, M., & Li, G. K. H. (2009). The symptom cluster of fatigue, pain, anxiety, and depression and the effect on the quality of life of women receiving treatment for breast cancer: a multicenter study. *Oncology Nursing Forum*, *36*(4). https://doi.org/10.1188/09.ONF.E205-E214

Statista (2020). *Number of smartphone users from 2016 to 2021(in billions).* https://www.statista.com/statistics/330695/number-of-smartphone-users-worldwide/

Statista (2021). *Number of mobile connections in Nigeria 2017-2021.* https://www.statista.com/statistics/1176097/number-of-mobile-connections-nigeria/

Stein, K. F., Sargent, J. T., & Rafaels, N. (2007). Intervention research: Establishing fidelity of the independent variable in nursing clinical trials. *Nursing Research, 56*(1), 54-62.

Sterne, J. A. C., Savović, J., Page, M. J., Elbers, R. G., Blencowe, N. S., Boutron, I., … Higgins, J. P. T. (2019). RoB 2: A revised tool for assessing risk of bias in randomised trials. *The BMJ*, *366*, 1–8. https://doi.org/10.1136/bmj.l4898

Stevens, D.J., Jackson, J.A., Howes, N., Morgan, J. (2014). Obesity Surgery Smartphone Apps: A Review. *Obesity Surgery 24*, 32–36. https://doi.org/10.1007/s11695-013-1010-3

Strauss, A., & Corbin, J. (1998). *Basics of qualitative research*: *Techniques and procedures for developing grounded theory.* Thousand Oaks, CA: Sage.

Swaminath *G. (2009).* Psychoeducation. *Indian journal of psychiatry, 51*(3), 171–172. https://doi.org/10.4103/0019-5545.55082

Swanson, J. M., Dibble, S. L., & Chapman, L. (1999). Effects of psycho-educational interventions on sexual health risks and psycho-social adaptation in young adults with genital herpes. *Journal of Advanced Nursing, 29(*4), 840–851. https://doi.org/10.1046/j.1365-2648.1999.00958.x

Thomas, D.R. (2003). *A general inductive approach for qualitative data analysis*. School of Population Health, University of Auckland. https://citeseerx.ist.psu.edu/viewdoc/download?doi=10.1.1.462.5445&rep=rep1&type=pdf

Urbaniak, G. C., & Plous, S. (2013). Research Randomizer (Version 4.0) [Computer software]. http://www.randomizer.org/

Vanderpuye, V., Grover, S., Hammad, N., PoojaPrabhakar, Simonds, H., Olopade, F., & Stefan, D. C. (2017). An update on the management of breast cancer in Africa. *Infectious Agents and Cancer, 12*(1), 13. https://doi.org/10.1186/s13027-017-0124-y

Wallace, B., & Kernozek, T. (2017). Self-efficacy theory applied to undergraduate biomechanics instruction. *Journal of Hospitality, Leisure, Sport and Tourism Education, 20,* 10–15. https://doi.org/10.1016/j.jhlste.2016.11.001

Watkins, C. C., Kanu, I. K., Hamilton, J. B., Kozachik, S. L., & Gaston-Johansson, F. (2017). Differences in coping among african American women with breast cancer and triple-negative breast cancer. *Oncology Nursing Forum, 44*(6), 689–702. https://doi.org/10.1188/17.ONF.689-702

Whitaker, R. (2012) Issues in mHealth: Findings from Key Informant Interviews. *Journal of Medical Internet Research 14*(5): e129. https://www.ncbi.nlm.nih.gov/pmc/articles/PMC3510768/

Wildenbos, G. A., Peute, L., & Jaspers, M. (2018). Aging barriers influencing mobile health usability for older adults: A literature based framework (MOLD-US). *International journal of medical informatics,* *114* 66–75. https://doi.org/10.1016/j.ijmedinf.2018.03.012

Wongpakaran, T., Wongpakaran, N., & Ruktrakul, R. (2011). Reliability and Validity of the Multidimensional Scale of Perceived Social Support (MSPSS): Thai Version. Clinical practice and epidemiology in mental health : *CP & EMH, 7*, 161–166. https://doi.org/10.2174/1745017901107010161

World Health Organization. (2018). mHealth, use of appropriate digital technologies for public health. *Seventy-First World Health Assembly - Provisional Agenda Item 12.4 (A71/20)*, *28*(March), 1–5. https://doi.org/10.2337/dc11-0366.4

World Health Organization (2019). WHO | Breast cancer. In *Who*. https://www.who.int/cancer/prevention/diagnosis-screening/breast-cancer/en/

World Health Organization (2020). Burden of Cancer- Nigeria's Cancer Burden Profile 2020 https://www.who.int/cancer/country-profiles/NGA_2020.pdf?ua=1

World Health Organization (2021). Breast Cancer. https://www.who.int/news-room/fact-sheets/detail/breast-cancer

World Medical Association (2013). World Medical Association Declaration of Helsinki: ethical principles for medical research involving human subjects. *JAMA, 310* (20), 2191–2194. https://doi.org/10.1001/jama.2013.281053

Wu, P.H., Chen,S.W., Huang, W.T.,Chang,S.C., &Hsu,M.C. (2018). Effects of a psychoeducational intervention in patients with breast cancer undergoing chemotherapy. *The Journal of Nursing Research, 26*(4), 266Y279. https://doi.org/10.1097/ jnr.0000000000000252

Xiao, W., Chow, K. M., So, W. K., Leung, D. Y., & Chan, C. W. (2016). The Effectiveness of Psychoeducational Intervention on Managing Symptom Clusters in Patients With Cancer: A Systematic Review of Randomized Controlled Trials. *Cancer Nursing, 39*(4), 279–291. https://doi.org/10.1097/NCC.0000000000000313

Zhang, Q., Zhang, L., Yin, R., Fu, T., Chen, H., & Shen, B. (2018). Effectiveness of telephone-based interventions on health-related quality of life and prognostic outcomes in breast cancer patients and survivors—A meta-analysis. *European Journal of Cancer Care*, *27*(1), 1–10. https://doi.org/10.1111/ecc.12632

Zhu, J., Ebert, L., & Chan, S. W. C. (2017). Integrative review on the effectiveness of internet-based interactive programs for women with breast cancer undergoing treatment. *Oncology Nursing Forum*, *44*(2), E42–E54. https://doi.org/10.1188/17.ONF.E42-E54

Zhu, J., Ebert, L., Liu, X., Wei, D., & Chan, S. W. C. (2018). Mobile breast cancer e-support program for chinese women with breast cancer undergoing chemotherapy (Part 2): Multicenter randomized controlled trial. *JMIR MHealth and UHealth*, *6*(4). https://doi.org/10.2196/mhealth.9438

Zimet, G.D., Dahlem, N.W., Zimet, S.G. & Farley, G.K. (1988). The Multidimensional Scale of Perceived Social Support. *Journal of Personality Assessment, 52*, 30-41.

Zulkosky, K. (2009). Self-Efficacy: A Concept Analysis. *Nursing Forum*, *44*(2), 93–102. https://doi.org/10.1111/j.1744-6198.2009.00132.x
