# Supplementary material for: Development and feasibility of an mHealth intervention for psychoeducational support of Nigerian women diagnosed with breast cancer undergoing chemotherapy: A pilot randomized controlled trial
Source: PLoS One. 2024 Nov 25;19(11):e0314365. doi: 10.1371/journal.pone.0314365 (PMC11588262; doi:10.1371/journal.pone.0314365)
Supplement: S2 File — (PDF) [file pone.0314365.s004.pdf]

Request for Permission to Publish Content under CC-BY License

Dear Rights Holder or Representative,

I have submitted a paper for publication in a PLOS journal, and wish to include the content listed below in the paper. I'm hereby requesting your (or your company's or institution's) permission to include the content in my paper. Please note that all PLOS journals are published under a Creative Commons Attribution License (CC BY), which allows for unrestricted use and distribution, even commercial, as long as attribution is given to the creator or rights holder of the content. See https://creativecommons.org/licenses/by/4.0/.

To grant me permission to use the content in my PLOS paper, please fill in the information below and then scan the completed form and send it to me at my email address.

Thank you.

My name:

Dr Akingbade Oluwadamilare

My email address:

oakingbade@link.cuhk.edu.hk

Description of the content which I'm seeking permission to use (citation and/or title, and pasted screen shot, if applicable):

I am seeeking permission to use the CENBA design figure from the Chemo Nurse Breast Cancer Application in a journal publication.

Link to the Content:

* * *

On behalf of myself or the rights holder, I hereby grant the permission sought herein.

Signature of Party Granting Permission:

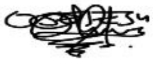

Date:

December 11, 2023

Printed Name and Title:

Adeagbo John Kehinde
Chief Executive Officer
Deltree Technologies
